# Functional characterization of a novel *p*-coumarate 3-hydroxylase from *Trametes versicolor*

**DOI:** 10.1128/aem.02301-25

**Published:** 2026-01-30

**Authors:** Link Hamajima, Reini Mori, Ryoga Tsurigami, Yuki Yoshida, Hiroyuki Kato, Mika Hayasaka, Hiromitsu Suzuki, Masashi Kato, Motoyuki Shimizu

**Affiliations:** 1 Faculty of Agriculture, Meijo University 12942, Nagoya, Japan; Shanghai Jiao Tong University, Shanghai, China

**Keywords:** caffeic acid, lignin, *Trametes versicolor*, *p*-coumarate 3-hydroxylase, flavoprotein monooxygenase

## Abstract

Lignin degradation by white-rot fungi proceeds through oxidative depolymerization of lignin polymers, followed by metabolism of the resulting low-molecular-weight aromatic fragments. Among these, the catabolism of the hydroxyphenyl (H) unit of *p*-coumaric acid (*p*-CA) remains poorly understood in fungi. Here, we investigated the metabolism of *p*-CA by *Trametes versicolor*. Two group A flavoprotein monooxygenases (FPMOs), *Tv*MNX3 and *Tv*MNX4, catalyzed the hydroxylation of *p*-CA to caffeic acid. Recombinant expression and biochemical analyses revealed that *Tv*MNX4 exhibited the highest catalytic efficiency toward both *p*-CA and 4-hydroxybenzoic acid, suggesting that it plays a major role in these hydroxylation reactions. Both enzymes also hydroxylated several *p*-CA derivatives, including 4-hydroxybenzaldehyde and 4-hydroxybenzyl alcohol, as well as other lignin-derived guaiacyl and syringyl compounds. Structural modeling and docking analyses indicated that the substrate-binding pocket—particularly residue Leu219 in *Tv*MNX4—is critical for substrate accommodation and catalytic activity. Together, these findings suggest that *T. versicolor* degrades *p*-CA via hydroxylation mediated by group A FPMOs. To our knowledge, this is the first report identifying *p*-CA 3-hydroxylase activity in eukaryotic FPMOs, expanding our understanding of fungal aromatic catabolism.

**IMPORTANCE:**

White-rot fungi are key players in the global carbon cycle through lignin degradation, yet the intracellular pathways that catabolize lignin-derived aromatics remain largely unresolved. The hydroxyphenyl unit compound *p*-coumaric acid (*p*-CA) is a major lignin fragment, but the enzymes responsible for its conversion to caffeic acid (CFA) have not been previously identified in fungi. This study demonstrates that *Trametes versicolor* employs group A flavoprotein monooxygenases (FPMOs) *Tv*MNX3 and *Tv*MNX4 for the hydroxylation of *p*-CA and related metabolites, representing an unrecognized branch of the *p*-CA catabolic pathway. Beyond ecological significance, the capacity of *Tv*MNX4 to generate bioactive phenolics such as CFA and piceatannol underscores its potential for biotechnological applications, including the sustainable synthesis of pharmaceuticals and polymer precursors.

## INTRODUCTION

Lignin is a randomly structured, heterogeneous phenylpropanoid polymer and one of the most abundant and recalcitrant biomaterials on Earth (1, 2). The degradation of lignin plays a key role in the carbon cycle of the biosphere, with white-rot basidiomycetes responsible for its complete mineralization (2–5). *Trametes versicolor*, a well-studied white-rot basidiomycete, degrades lignin through a unique enzymatic system comprising lignin peroxidases, manganese peroxidases, and laccases (3–5). These extracellular enzymes act as nonspecific one-electron oxidants that attack the lignin polymer (4–7), generating various low-molecular-weight aromatic fragments (8, 9). These aromatic fragments are taken up by fungal cells and further metabolized through specific oxidative and ring-cleavage pathways (10).

Lignin-derived fragments are key intermediates in lignin biodegradation by white-rot fungi and can be classified into three structural units based on their chemical features (11): (i) guaiacyl (G), such as vanillic acid (VA) and ferulic acid (FA); (ii) syringyl (S), such as syringic acid and sinapic acid; and (iii) hydroxyphenyl (H), such as 4-hydroxybenzoic acid (4-HBA) and *p*-coumaric acid (*p*-CA) (12). These fragments are subsequently oxidized, decarboxylated, hydroxylated, and/or demethoxylated intracellularly to yield 1,2,4-trihydroxybenzene (THB) and/or methoxyhydroquinone (MHQ), which are then subjected to aromatic ring cleavage catalyzed by dioxygenases (13). Among these lignin-derived fragments, the degradation of the hydroxyphenyl unit (H-unit) compound *p*-CA has been proposed to proceed through two main pathways (14–16): (i) a β-oxidation-like reductive pathway leading to the formation of 4-HBA and/or 4-hydroxybenzaldehyde (4-HBALD), and (ii) a ring-hydroxylating reaction that produces caffeic acid (CFA). *p*-CA is one of the major phenolic acids released during lignin degradation; thus, elucidating its fungal hydroxylation mechanism is crucial for understanding aromatic catabolism. However, the enzymes responsible for the 3-hydroxylation of *p*-CA to CFA in pathway (ii) remain largely obscure in white-rot fungi (16).

CFA is a naturally occurring phenolic compound with diverse biological activities, including antioxidant (17, 18), antiviral (19), anticancer (20), and anti-inflammatory effects (21). Recently, phenylpropanoic acids such as CFA have garnered increasing interest due to their broad pharmaceutical potential and value as monomers in liquid crystal polymer synthesis for electronic applications (22–24). Given these bioactive and industrially relevant properties, fungal *p*-CA hydroxylases that catalyze the formation of CFA may serve as promising biocatalysts for the sustainable production of high-value phenolics.

In many plant species, *p*-CA 3-hydroxylases (UniProt ID: O22203) belonging to the cytochrome P450 superfamily have been identified (25–27). These enzymes catalyze the 3′-hydroxylation of *p*-coumaric esters of shikimic and quinic acids, generating intermediates of lignin phenylpropanoid biosynthesis, such as CFA derivatives (25–27). In contrast, plant species lacking caffeoyl shikimate esterase, such as *Zea mays* and *Brachypodium distachyon*, possess distinct *p*-CA 3-hydroxylases (UniProt IDs: B6U9S6, I1H6P1) that are structurally related to ascorbate peroxidases and utilize heme as a cofactor (28). In certain bacteria, *p*-CA 3-hydroxylase from *Saccharothrix espanaensis* (UniProt ID: Q2EYY8) and 4-hydroxyphenylacetate 3-hydroxylase from *Escherichia coli* (UniProt ID: Q57160)—both classified as flavoprotein monooxygenases (FPMOs)—catalyze the hydroxylation of *p*-CA to produce CFA (26, 27). Currently, FPMOs are divided into eight groups (A–H) based on distinct structural and functional properties (29). Among these, the FPMOs responsible for *p*-CA hydroxylation in bacteria belong to group D, which consists of a two-component enzyme system comprising a reductase and an oxygenase (26, 27). Although several *p*-CA 3-hydroxylases have been well characterized, in plants and bacteria, the enzymatic mechanisms responsible for *p*-CA hydroxylation in fungi—particularly in white-rot basidiomycetes—remain largely unexplored.

The genome sequence of *T. versicolor* reveals a high degree of diversity among FPMOs, with 41 FPMO-related genes identified (https://mycocosm.jgi.doe.gov/trave1/trave1.home.html). According to the FPMO family classification based on conserved sequence fingerprints (groups A–H) proposed by Paul et al. (29), 29 and 12 of these genes encode group A and group B FPMOs, respectively. Notably, no group D FPMOs, enzymes known to catalyze *p*-CA hydroxylation in bacteria, are present in the genome (Fig. S1A). Several *T. versicolor* FPMOs have previously been characterized at the biochemical and structural levels. *Tv*MNX3 (protein ID: 58730) has been identified as a hydroquinone (HQ) 2-hydroxylase, and its crystal structure has been resolved (PDB ID: 8R2U) (16, 30). In addition, *Tv*MNX1 (protein ID: 175239) functions as a 4-hydroxybenzoate 1-hydroxylase (decarboxylase), catalyzing the oxidative decarboxylation of lignin-derived aromatic compounds such as 4-HBA and VA in *T. versicolor* (30). Considering that several FPMOs in group A catalyze aromatic hydroxylation in bacteria and fungi, it is plausible that certain *T. versicolor* FPMOs also participate in *p*-CA hydroxylation (10).

Therefore, in this study, we sought to elucidate the enzymatic basis of *p*-CA hydroxylation in *T. versicolor* by heterologously producing and characterizing group A FPMOs, including the well-studied *Tv*MNX1 and *Tv*MNX3. The findings were analyzed in the context of enzymatic degradation of *p*-CA by *T. versicolor*.

## RESULTS

### Fungal metabolism of *p*-CA

**Fig 1:**
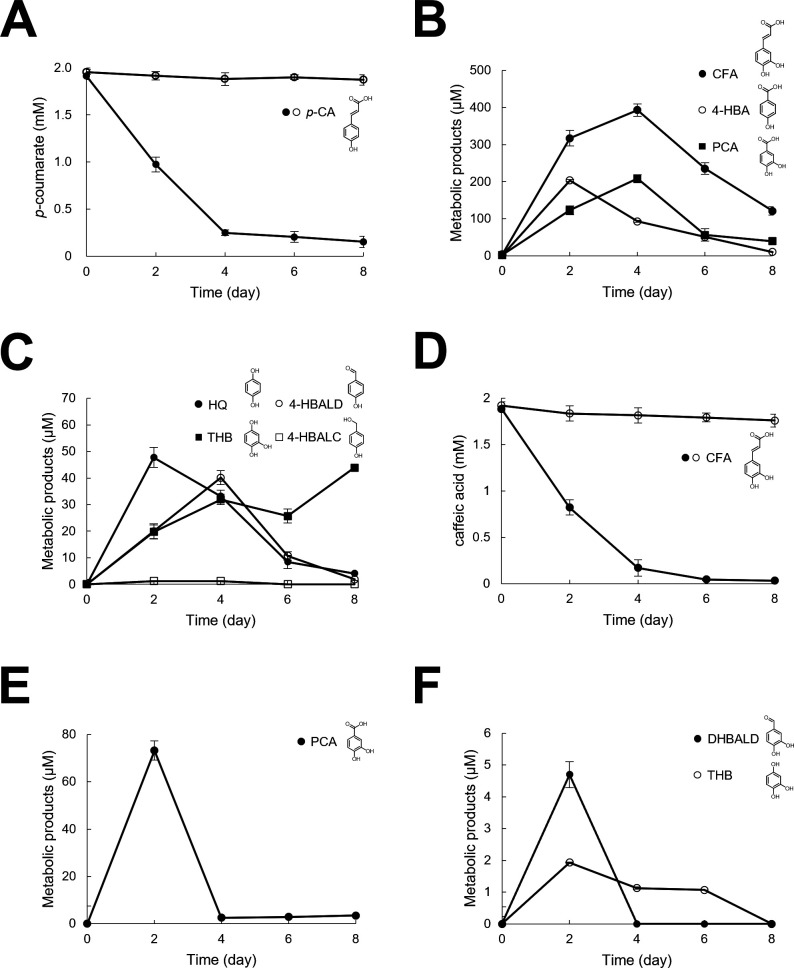
Fungal metabolism of *p*-CA. (**A**) Time course of *p*-CA conversion. After a 6 day preincubation, *p*-CA (●) was added to a final concentration of 2.0 mM; 〇, uninoculated control. (**B, C**) Metabolites of *p*-CA were identified using gas chromatography–mass spectrometry (GC–MS) with authentic standards as references. After 2 days of incubation with *p*-CA, the following metabolites were detected: (**B**) CFA (●); 4-HBA (〇); PCA (■); and (**C**) HQ (●); 4-HBALD (〇); THB (■); 4-HBALC (□). (**D**) Time course of CFA conversion. After a 6 day preincubation, CFA (●) was added to a final concentration of 2.0 mM; 〇, uninoculated control. (**E, F**) Metabolites of CFA were identified using GC–MS with reference to authentic standards after 2 days of incubation. (**E**) PCA produced from CFA (●). (**F**) DHBALD, (●); THB (〇). Data represent the mean ± standard deviation of three independent experiments.

Evaluation of *p*-CA metabolism by *T. versicolor* revealed a decline in its concentration from an initial 2.0 mM to 0.15 mM after 8 days of culture (Fig. 1A; Fig. S2A through D). Mono-hydroxylated products—4-HBA, 4-HBALD, and 4-hydroxybenzyl alcohol (4-HBALC)—and di-hydroxylated products—CFA, protocatechuic acid (PCA), HQ, and THB—were identified as fungal metabolites of *p*-CA (Fig. 1B and C). The same *p*-CA–derived metabolites were previously reported (16), and that study suggested that *p*-CA is metabolized via a CoA-dependent β-oxidation pathway to 4-HBA and/or 4-HBALD in *T. versicolor*. Our results indicate that *p*-CA is converted by *T. versicolor* through two distinct routes: a CoA-dependent β-oxidation route yielding 4-HBALD and/or 4-HBA (16), and an ortho-hydroxylation route producing CFA. Next, we monitored the metabolism of CFA to evaluate its subsequent conversion by *T. versicolor*. The CFA concentration decreased from an initial 2.0 mM to 0.03 mM in the medium after 8 days of cultivation, indicating that the substrate was further degraded by *T. versicolor* (Fig. 1D; Fig. S2E and F). Additionally, PCA, 3,4-dihydroxybenzaldehyde (DHBALD), and THB were identified as fungal metabolites of CFA (Fig. 1E and F). The proposed metabolic pathways of *p*-CA and CFA are summarized in Fig. S3. In this study, we further aimed to identify the *T. versicolor* proteins responsible for hydroxylating *p*-CA to CFA.

### Search for a *p*-CA 3-hydroxylase from the *T. versicolor* genome

As mentioned above, the *T. versicolor* genome encodes 41 FPMO genes, comprising 29 group A and 12 group B enzymes (Fig. S1A). Among these, 4-hydroxybenzoate 1-hydroxylase (decarboxylase) *Tv*MNX1 and HQ 2-hydroxylase *Tv*MNX3 were previously identified (16, 30). A phylogenetic analysis of biochemically characterized group A FPMOs has shown that enzymes acting on 4-HBA, such as 4-hydroxybenzoate 3-hydroxylase (4HB3H and PHBH) and 4-hydroxybenzoate 1-hydroxylase (4HB1H), are distributed across three distinct clades (31). Moreover, 4HB3H from *Aspergillus niger* (PhhA; UniProt ID: A2QGH7) clusters with phenol hydroxylase from *Trichosporon cutaneum* (PHHY; UniProt ID: P15245), HQ hydroxylase (HQH) from *Candida parapsilosis* CBS604 (HQH; UniProt ID: G8BGH1), and 3-hydroxybenzoate 4-hydroxylase (3HB4H) from *Comamonas testosteroni* (mobA; UniProt ID: Q6SSJ6). Consistent with this classification (Fig. S1B), *Tv*MNX3 was located within the same clade as PhhA, PHHY, HQH, and 3HB4H, suggesting that the enzyme possesses an additional thioredoxin-like domain characteristic of this subgroup of group A FPMOs. By contrast, *Tv*MNX1 clustered with 4HB1H from *C. parapsilosis* (CpMNX1; UniProt ID: G8B709) and prenyl-4-hydroxybenzoate decarboxylase (VibMO1; UniProt ID: A0A167KUL3).

**Fig 2:**
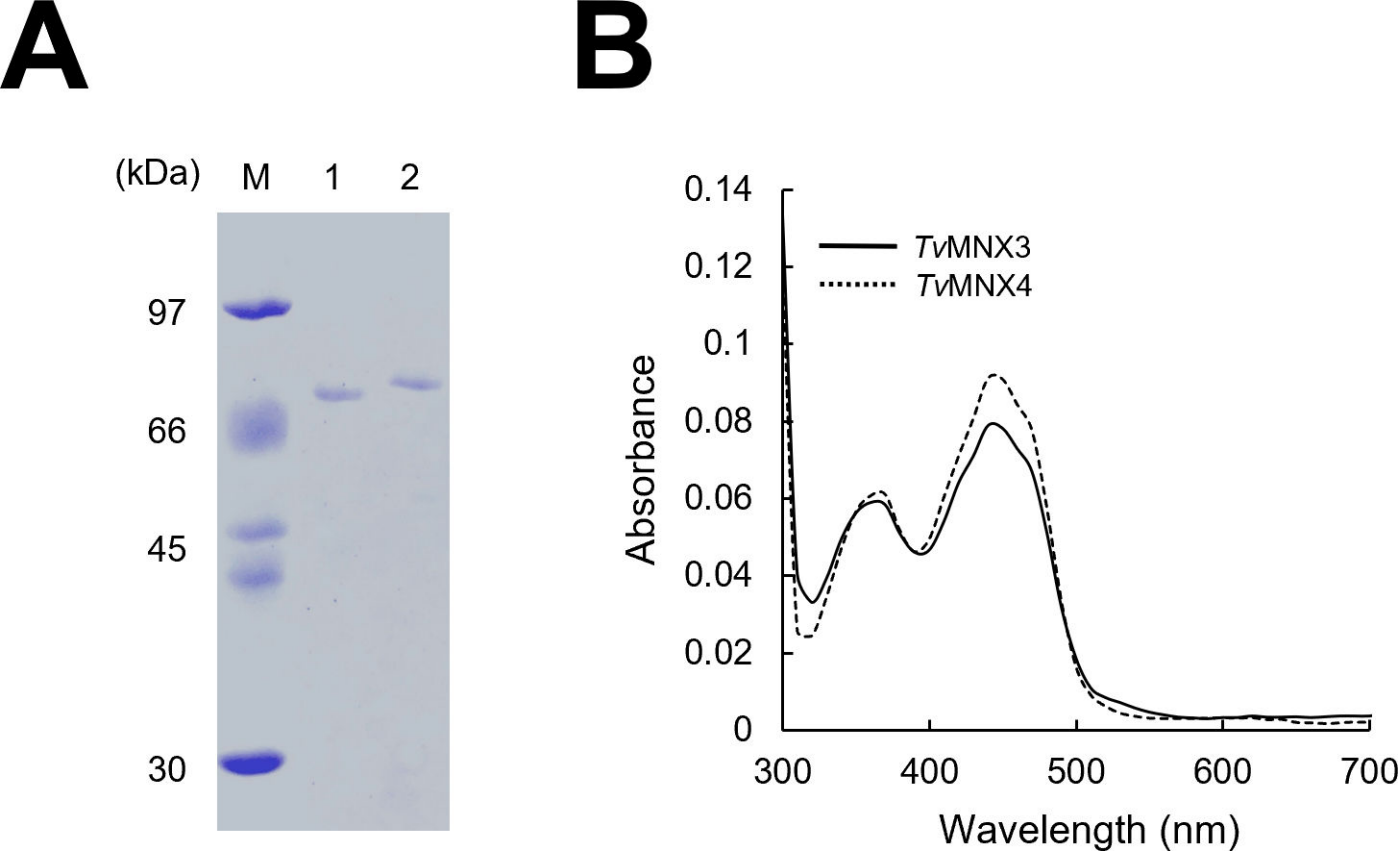
SDS-PAGE analysis and absorption spectra of recombinant *Tv*MNX3 and *Tv*MNX4. (**A**) SDS-PAGE analysis of purified recombinant proteins. Lane 1, *Tv*MNX3; lane 2, *Tv*MNX4; lane M, molecular-mass marker. (**B**) UV-visible absorption spectra of purified *Tv*MNX3 and *Tv*MNX4.

To identify a *p*-CA 3-hydroxylase, we selected seven *fpmo* genes representing distinct phylogenetic clades across group A and B FPMOs (Fig. S1B and C). The selection was based on phylogenetic trees constructed separately for *T. versicolor* FPMOs and for functionally characterized group A and B FPMOs using the neighbor-joining method with 1,000 bootstrap replicates (Fig. S1B and C). Although group B FPMOs have not been reported to catalyze aromatic hydroxylation reactions (29), representative group B genes were included in the initial selection to ensure broad functional coverage and to experimentally assess whether any atypical activities might exist in *T. versicolor*. Representative sequences were chosen from each major clade to encompass the overall sequence diversity. Although the *p*-CA 3-hydroxylase activity could not be precisely predicted from the primary amino acid sequences alone, seven candidates were selected—group A: *Tv*MNX1, *Tv*MNX3, *Tv*47635, *Tv*32834, *Tv*48947; group B: *Tv*55900, *Tv*74154—which clustered with biochemically characterized enzymes such as 4HB1H, PHHY, HQH, salicylate hydroxylase (UniProt ID: A0A1M2V9Y2), hispidin-3-hydroxylase (h3h: UniProt ID: A0A3G9K5C8), indole-3-pyruvate monooxygenase (YUC8; UniProt ID: A0A1C7MSE3), and thiol-specific monooxygenase (FMO1; UniProt ID: A0A1C7LSE7) (Fig. S1B and C). We prepared the seven FPMOs using an *E. coli* expression system (Fig. 2A; Fig. S4A).

The C-terminal 6×His-tagged candidate enzymes were produced in *E. coli* for biochemical characterization (Fig. 2A; Fig. S4A). SDS-PAGE analysis (Fig. S4A) showed single major bands with apparent molecular masses closely matching theoretical values predicted from their amino acid sequences (Table S1). The UV-visible absorption spectra of the purified recombinant FPMOs exhibited characteristic flavoprotein features, with absorption maxima at 380 and 450 nm (Fig. 2B; Fig. S4B). Theoretical and experimental A280/A450 ratios are summarized in Table S1. Ratios close to the theoretical values (≈5.13–11.8) indicated that all enzymes were predominantly in the holo form, with full or near-full occupancy of the bound flavin adenine dinucleotide (FAD) cofactor (Table S1).

### Catalytic conversion of *p*-CA derivatives

**Fig 3:**
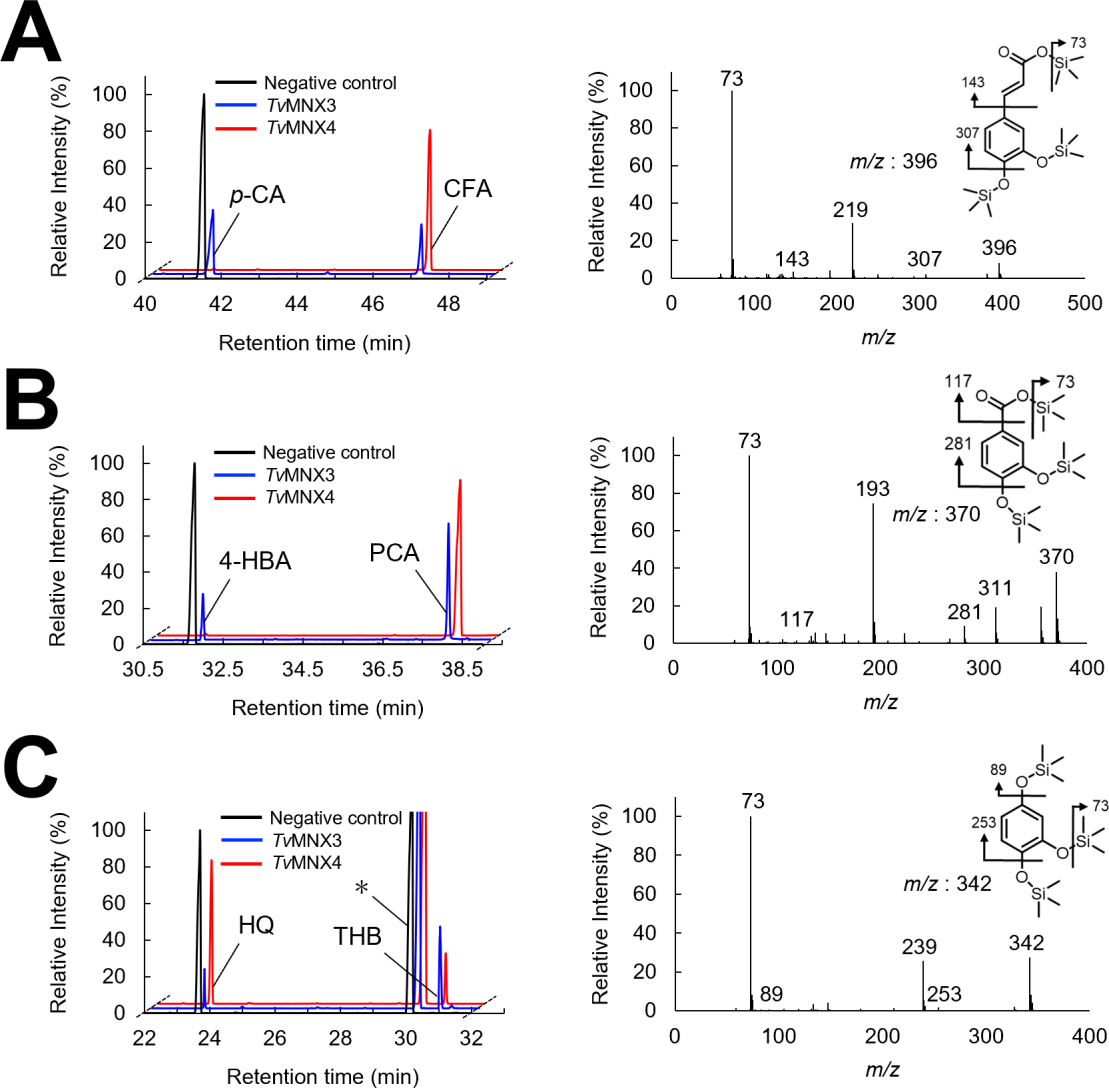
Total ion chromatograms and mass spectra of reaction products generated by *Tv*MNX3 and *Tv*MNX4. Reaction products generated from *p*-CA (**A**), 4-HBA (**B**), and HQ (**C**) were analyzed. The trimethylsilyl (TMS)-derivatized products were analyzed by GC–MS. Mass spectra of the reaction products—(**A**) CFA, (**B**) PCA, and (**C**) THB—were obtained from GC peaks at retention times of 47.0 min (**A**), 38.0 min (**B**), and 30.8 min (**C**). Asterisks indicate contaminants. Each experiment was performed three times, and representative data are shown.

The catalytic conversion of *p*-CA was analyzed, using NADPH and NADH as cosubstrates, to evaluate the enzymatic activities of the seven recombinant FPMOs. The hydroxylated products of *p*-CA were identified by gas chromatography–mass spectrometry (GC–MS) analysis (Fig. 3A; Fig. S5). The mass spectra of the trimethylsilyl (TMS) derivatives of the reaction products showed fragmentation patterns identical to those of CFA (Fig. 3A). Among the seven recombinant FPMOs, only *Tv*MNX3 and *Tv*47635 (hereafter referred to as *Tv*MNX4) were capable of hydroxylating *p*-CA to CFA (Fig. 3A; Fig. S5). Additionally, *Tv*MNX3 exhibited activity only with NADPH, whereas *Tv*MNX4 utilized both NADPH and NADH, showing higher catalytic efficiency with NADPH. This is the first study to identify and characterize *p*-CA 3-hydroxylase activity among members of the group A FPMO superfamily. *Tv*MNX3 was previously shown to hydroxylate HQ and 4-HBA to THB and PCA, respectively (9, 30). When tested under identical conditions, *Tv*MNX4 also catalyzed the hydroxylation of HQ and 4-HBA (Fig. 3B and C).

**Fig 4:**
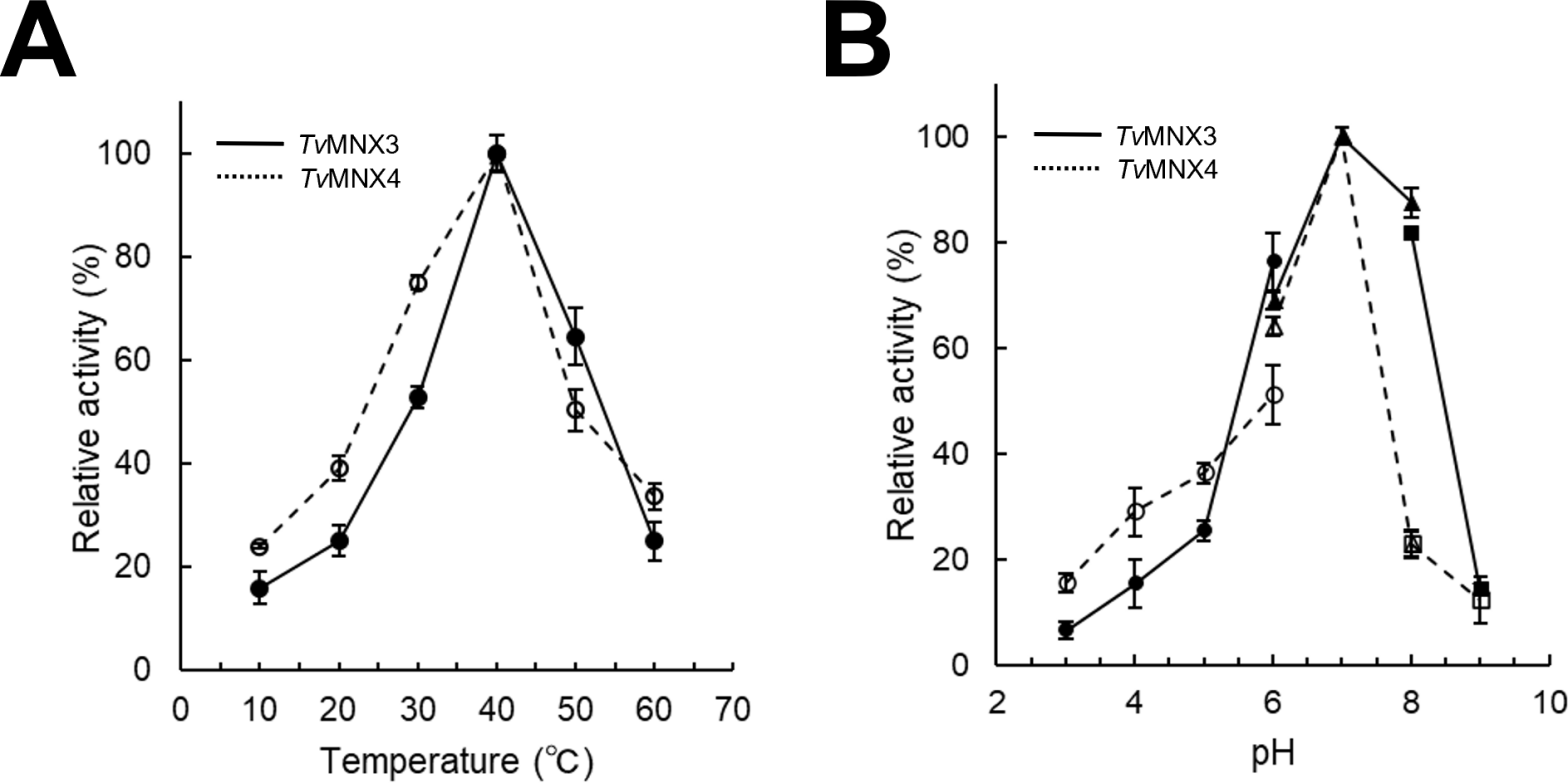
Temperature and pH optima of *Tv*MNX3 and *Tv*MNX4. (**A**) Temperature dependence of *Tv*MNX3 and *Tv*MNX4 activities using HQ and 4-HBA as substrates, respectively. Reactions were conducted at temperatures ranging from 10°C to 60°C. (**B**) pH dependence of *Tv*MNX3 and *Tv*MNX4 activities over the pH range 3.0–9.0 using 50 mM sodium acetate (pH 3.0–6.0; ●), 50 mM HEPES-NaOH (pH 6.0–8.0; ▲), and 50 mM Tris-HCl (pH 8.0–9.0; ■) buffers. Data are presented as mean ± standard deviation of three independent experiments.

Subsequently, phenol (PH) and 4-HBA derivatives were tested as substrates for *Tv*MNX3 and *Tv*MNX4. The hydroxylated products of PH, 4-HBALD, and 4-HBALC were identified by GC–MS (Fig. S6A through C). Both *Tv*MNX3 and *Tv*MNX4 hydroxylated 4-HBALD and 4-HBALC to DHBALD and 3,4-dihydroxybenzyl alcohol (DHBALC), respectively, whereas only *Tv*MNX4 converted PH to catechol (CAT). Next, we examined G (G-unit) and S (S-unit) lignin-derived fragments, including MHQ, FA, and 2,6-dimethoxyhydroquinone (DMHQ), as well as diphenolic compound (stilbenoid) resveratrol (RES). *Tv*MNX3 and *Tv*MNX4 converted MHQ to its hydroxylated product, while only *Tv*MNX4 hydroxylated DMHQ, FA, and RES (Fig. S6D through G). Overall, *Tv*MNX3 and *Tv*MNX4 catalyzed the conversion of 6 and 10 *p*-CA derivatives and their metabolic products, respectively (Fig. S7). The optimal reaction conditions for *Tv*MNX3 and *Tv*MNX4 activities were determined using HQ and *p*-CA as substrates, respectively, and were found to be 40°C and pH 7.0 (Fig. 4A and B).

### Hydroxylation efficiencies and kinetic parameters

Hydroxylation efficiency represents a key measure of catalytic performance in flavin-dependent monooxygenases in group A FPMOs, reflecting how effectively reducing equivalents from NADPH are used for substrate hydroxylation rather than for uncoupled oxygen reduction (32). Following this concept, the hydroxylation efficiencies of *Tv*MNX3 and *Tv*MNX4 were determined by monitoring NADPH oxidation at 340 nm and quantifying product formation by GC–MS. *Tv*MNX3 exhibited the highest hydroxylation efficiency toward HQ (90%), followed by 4-HBA (67%), 4-HBALD (40%), and *p*-CA (22%) (Table 1). In contrast, *Tv*MNX4 displayed a broader substrate specificity, with particularly high efficiencies toward *p*-CA (94%) and 4-HBA (92%), whereas its hydroxylation efficiencies toward other aromatic compounds—HQ, 4-HBALD, 4-HBALC, MHQ, and RES—were moderate (29%, 34%, 22%, 20%, and 38%, respectively).

**TABLE 1:** Kinetic parameters and hydroxylation efficiencies of *Tv*MNX3, *Tv*MNX4, and the *Tv*MNX4_L219R variant toward phenolic compounds^*a*^

Substrate	*Tv*MNX3				*Tv*MNX4				*Tv*MNX4_L219R			
	*K_m_* (µM)	*k_cat_* (s^−1^)	*k_cat_/K_m_* (s^−1^/µM)	Hydroxylation efficiency (%)	*K_m_* (µM)	*k_cat_* (s^−1^)	*k_cat_/K_m_* (s^−1^/µM)	Hydroxylation efficiency (%)	*K_m_* (µM)	*k_cat_* (s^−1^)	*k_cat_/K_m_* (s^−1^/µM)	Hydroxylation efficiency (%)
*p*-CA	71 ± 1.1	8.2 ± 0.71	0.12	22	43 ± 1.9	22 ± 0.54	0.52	94	337 ± 29	1.5 ± 0.090	0.0044	5.8
4-HBA	31 ± 0.91	13 ± 0.76	0.42	67	27 ± 0.84	13 ± 0.54	0.48	92	2.4 ± 0.18	2.5 ± 0.10	1.0	74
HQ	13 ± 1^*b*^	6.8 ± 0.1^*b*^	0.52^*b*^	90	222 ± 9.1	11 ± 0.65	0.050	29	3.5 ± 0.30	7.5 ± 0.41	2.1	96
PH	–	–	–	–	525 ± 12	3.5 ± 0.067	0.0066	5.6	71 ± 4.1	5.1 ± 0.24	0.072	21
4-HBALD	39 ± 1.4	6.3 ± 0.86	0.16	40	59 ± 1.2	5.3 ± 0.93	0.090	34	4.8 ± 0.35	1.72 ± 0.038	0.36	34
4-HBALC	126 ± 7.4	6.5 ± 1.1	0.052	14	159 ± 9.3	4.4 ± 0.25	0.028	22	20 ± 2.1	1.8 ± 0.18	0.090	18
MHQ	746 ± 16	4.7 ± 0.038	0.0062	7.0	174 ± 2.3	2.5 ± 0.11	0.014	20	–	–	–	–
DMHQ	–	–	–	–	731 ± 17	3.8 ± 0.070	0.0053	15	–	–	–	–
FA	–	–	–	–	848 ± 16	3.3 ± 0.065	0.0039	3.1	–	–	–	–
RES	–	–	–	–	151 ± 5.1	6.8 ± 1.3	0.045	38	–	–	–	–
NADH	–	–	–	–	275 ± 4.8	2.5 ± 0.61	0.0091	–	266 ± 5.2	1.1 ± 0.74	0.042	–
NADPH	47 ± 2.7	8.3 ± 0.62	0.18	–	41 ± 3.1	9.5 ± 0.80	0.23	–	49 ± 2.8	6.0 ± 1.7	0.12	–

^*a*^ The enzymatic activities of *Tv*MNX3, *Tv*MNX4, and *Tv*MNX4_L219R were determined in 0.5 mL reaction mixtures containing 0.1 μM enzyme and 10 μL of substrate solution (0 mM–600 mM in dimethyl sulfoxide) in 50 mM HEPES–NaOH buffer (pH 7.0) at 40°C. Initial velocities were measured for 30 s following substrate addition and used to calculate kinetic parameters. Data represent the mean ± standard deviation from four independent experiments. – indicates no detectable enzymatic activity.

^*b*^ Data for HQ were obtained from Kuatsjah et al. (30).

The kinetic parameters of *Tv*MNX3 and *Tv*MNX4 were investigated using *p*-CA and its derivatives as substrates (Table 1). *Tv*MNX3 was active with *p*-CA and several of its derivatives. The catalytic efficiency (*k*_cat_/*K*_m_) of *Tv*MNX3 for HQ was determined as described previously (30). Among the six tested substrates, the highest catalytic efficiency was observed for HQ. The lowest *K*_m_ value was also obtained for HQ, whereas the highest *k*_cat_ value was observed with 4-HBA. The *k*_cat_/*K*_m_ value for 4-HBA was 3.5-fold higher than that for *p*-CA. *Tv*MNX4 exhibited activity toward 10 substrates, with *p*-CA and 4-HBA showing the highest and comparable catalytic efficiencies (Table 1). The enzyme displayed a marginally lower *K*_m_ for 4-HBA and a marginally higher *k*_cat_ for *p*-CA, resulting in comparable overall *k*_cat_/*K*_m_ values for both substrates. The *k*_cat_/*K*_m_ values of *Tv*MNX3 for HQ, 4-HBALD, and 4-HBALC were higher than those of *Tv*MNX4, whereas the values of *Tv*MNX4 for *p*-CA, 4-HBA, and MHQ exceeded those of *Tv*MNX3 (Table 1). Consistent with their cofactor dependencies, *Tv*MNX3 utilized only NADPH, whereas *Tv*MNX4 accepted both NADPH and NADH, showing higher catalytic efficiency with NADPH (Table 1).

### Comparison of predicted structures

To compare the substrate specificities of *Tv*MNX3 and *Tv*MNX4 with other hydroxylases within the same group A FPMO clade (Fig. S1B), recombinant C-terminal 6×His-tagged PHHY from *T. cutaneum* (Fig. S8A and B) and *An*PhhA (UniProt ID: C8VBV0) from *A. nidulans* (Fig. S9A and B) were produced in *E. coli*. Multiple sequence alignment revealed that PHHY, PhhA from *A. niger*, *Tv*MNX3, and *Tv*MNX4 share several conserved amino acid residues, including the tyrosine and aspartate pairs responsible for substrate positioning (Fig. S10). However, the *phhA* gene from *A. niger* has been reported to be poorly expressed in *E. coli* BL21 cells (33). Therefore, we produced a PhhA homolog from *A. nidulans* (*An*PhhA), which shares 83.6% amino acid sequence identity with the *A. niger* enzyme (Fig. S1B). PHHY is known to function as a homodimeric enzyme, and *Tv*MNX3, *Tv*MNX4, and *An*PhhA were also observed to exist as dimers (Fig. S11). PHHY exhibited activity toward PH and its derivatives, with the highest *K*_m_ (3 µM) and *k*_cat_ (7.8 s⁻¹) observed for PH as the substrate (34, 35). The catalytic efficiency (*k*_cat_/*K*_m_) of PHHY for PH (2.6 s⁻¹ µM⁻¹) was markedly higher than that of *Tv*MNX3 (no detectable activity) and *Tv*MNX4 (0.0066 s⁻¹ µM⁻¹). PHHY has been reported to be inactive toward phenolic aromatic acids (36, 37). Accordingly, we evaluated whether *p*-CA could serve as a substrate for PHHY and *An*PhhA. GC–MS analysis showed that PHHY catalyzed the hydroxylation of PH to CAT (Fig. S8C), whereas no conversion products of *p*-CA were detected (Fig. S8D). By contrast, the reaction product PCA was significantly produced from 4-HBA by *An*PhhA (Fig. S9C), and *An*PhhA also catalyzed the hydroxylation of *p*-CA to form CFA (Fig. S9D). These findings indicate that FPMOs classified within the same clade can exhibit distinct substrate specificities toward *p-*CA.

**Fig 5:**
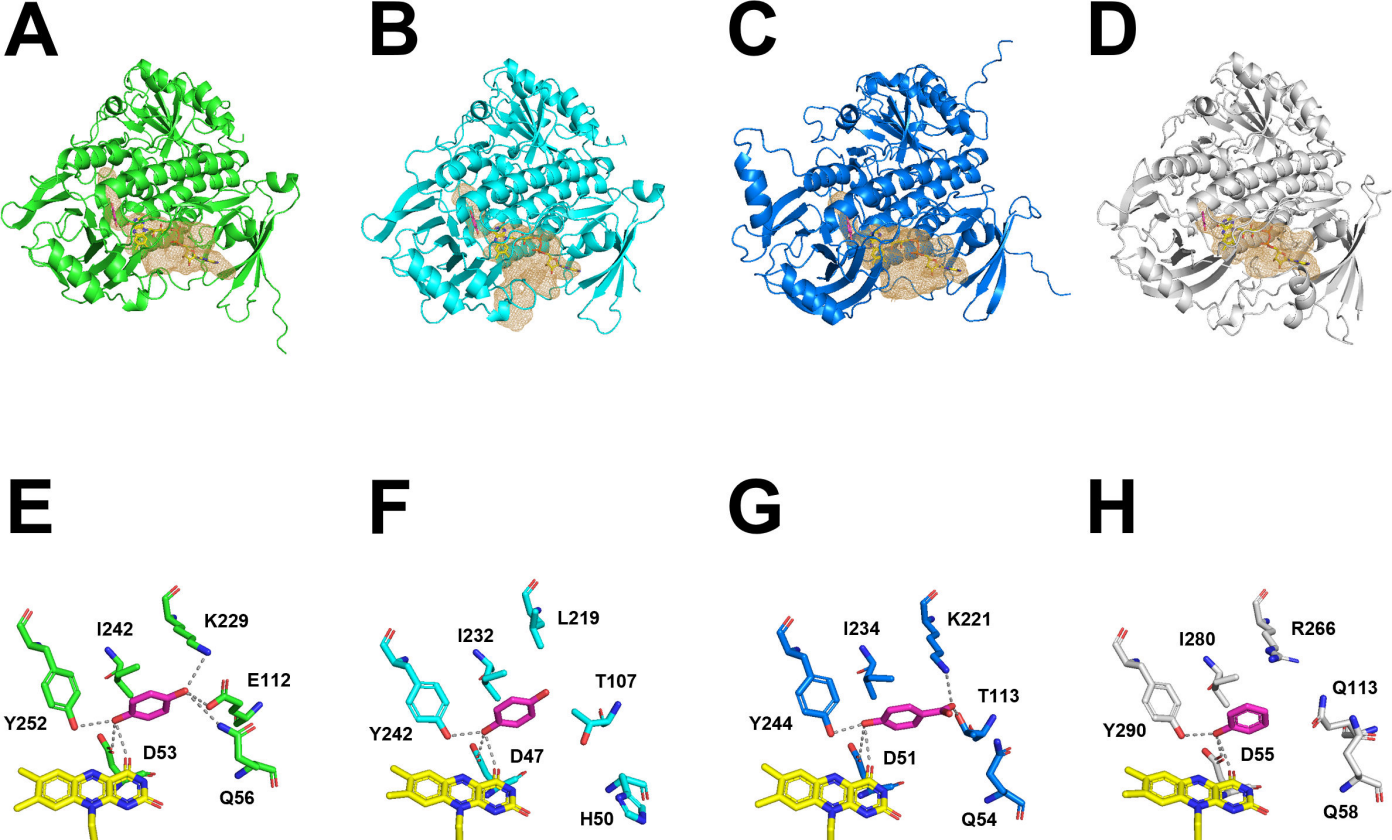
Structural comparison of *Tv*MNX3, *Tv*MNX4, *An*PhhA, and PHHY. (**A–D**) Overall structures of *Tv*MNX3, *Tv*MNX4, *An*PhhA, and PHHY. (**A–C**) Closed-conformation models of *Tv*MNX3, *Tv*MNX4, and *An*PhhA docked with HQ or 4-HBA, predicted using the GNINA docking program. (**D**) Crystal structure of PHHY liganded with PH (PDB ID: 1PN0). (E–H) Active sites of *Tv*MNX3 (**E**), *Tv*MNX4 (**F**), and *An*PhhA (**G**) docked with HQ or 4-HBA as substrates, showing FAD (yellow sticks) and substrates (magenta sticks); (**H**) active site of PHHY liganded with PH (PDB ID: 1PN0).

To elucidate the structural basis underlying the different substrate specificities toward *p*-CA, we generated and compared predicted three-dimensional models of *Tv*MNX3, *Tv*MNX4, *An*PhhA, and PHHY. Group A FPMOs are known to undergo conformational transitions between open and closed FAD states during catalysis (32, 38, 39). In the open conformation, the isoalloxazine ring of FAD is positioned to accept electrons from NAD(P)H, whereas in the closed conformation, it becomes properly aligned for oxygen activation and substrate hydroxylation (32, 38, 39). Crystal structures have been determined for *Tv*MNX3 (PDB ID: 8R2U), resolved in an open conformation with bound FAD, and PHHY (PDB ID: 1PN0), resolved in a closed conformation with bound FAD and liganded PH (30, 40). In addition, a docking model between the substrate HQ and *Tv*MNX3, along with a proposed reaction mechanism, has been reported (30). To complement these data, we generated predicted structures of *Tv*MNX3, *Tv*MNX4, and *An*PhhA with bound FAD in a closed conformation using AlphaFold2 (Fig. S12). We next employed the GNINA docking program to predict substrate-binding sites in the *Tv*MNX4 and *An*PhhA structures. As reported for the substrate-binding pockets of PHHY, HQH, 3HB4H, and *A. niger* PhhA (31), the resulting substrate-binding models displayed similar orientations among the three enzymes, suggesting that the predicted ligand positioning at the active site was accurate (Fig. 5A through D). Although the amino acid sequence identities between *Tv*MNX4 and PHHY, *Tv*MNX3, or *An*PhhA were 38.6%, 57.7%, and 36.1%, respectively, these proteins shared a highly conserved overall fold, the presence of a bound FAD cofactor, and a characteristic combination of α-helices and β-sheets within the substrate-binding domain (Fig. 5A through D). The root-mean-square deviations between the crystal structure of PHHY in the closed conformation and the predicted structures of *Tv*MNX3, *Tv*MNX4, and *An*PhhA were 1.252, 1.115, and 1.370, respectively, indicating structural similarity. Based on structural comparison with PHHY (41), the adjacent aspartate residues (Asp53 in *Tv*MNX3, Asp47 in *Tv*MNX4, and Asp51 in *An*PhhA) are proposed to contribute to the stabilization and activation of the flavin-oxygen intermediate (Fig. 5E through H; Fig. S10). The conserved tyrosine residues (Tyr252 in *Tv*MNX3, Tyr242 in *Tv*MNX4, and Tyr244 in *An*PhhA) are located in the active site and likely assist in substrate binding (Fig. 5E through H; Fig. S10).

**Fig 6:**
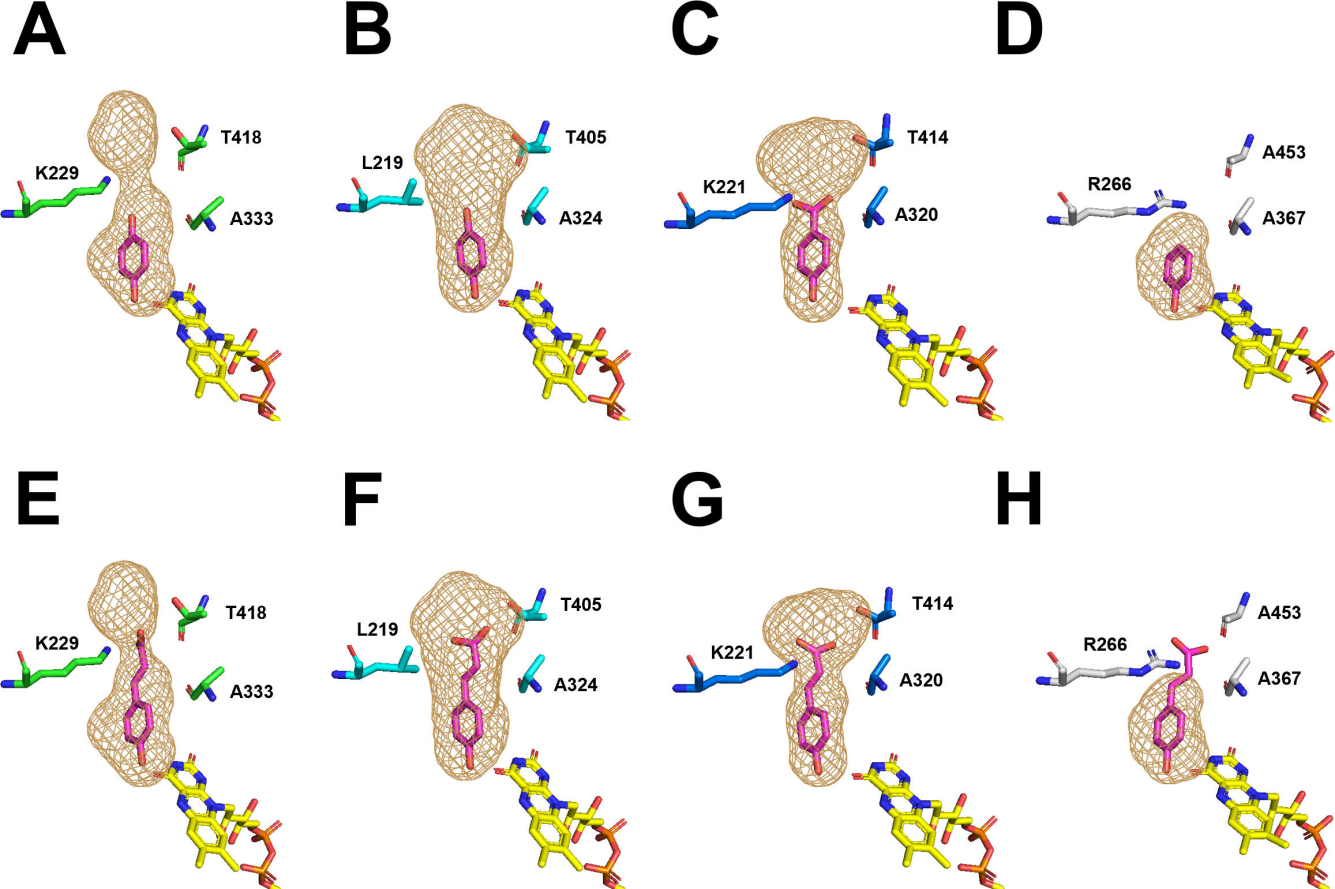
Comparison of cavities in the active-site pockets of *Tv*MNX3, *Tv*MNX4, *An*PhhA, and PHHY. Cavities in the active-site pockets of *Tv*MNX3 (**A, E**), *Tv*MNX4 (**B, F**), *An*PhhA (**C, G**), and PHHY (**D, H**) are shown as brown mesh surfaces. HQ or 4-HBA docked in the active-site pockets of *Tv*MNX3 (**A**), *Tv*MNX4 (**B**), *An*PhhA (**C**), and the PHHY crystal structure liganded with PH (PDB ID: 1PN0) (**D**). *p*-CA docked in the active-site pockets of *Tv*MNX3 (**E**), *Tv*MNX4 (**F**), *An*PhhA (**G**), and PHHY (**H**). Magenta and yellow sticks represent the substrates and FAD, respectively.

Additionally, we generated *p*-CA–binding models for the four FPMOs. When *p*-CA was positioned at the same location as HQ, 4-HBA, or PH, the substrates were well docked into the active-site pockets of *Tv*MNX3, *Tv*MNX4, and *An*PhhA (Fig. 6A through H). In contrast, *p*-CA could not be docked into the pocket of PHHY (Fig. 6H) because its active-site cavity was smaller than those of *Tv*MNX3, *Tv*MNX4, and *An*PhhA (Fig. 6E through H). These results indicate that the spatial architecture of the upper region of the active-site pocket in *Tv*MNX3, *Tv*MNX4, and *An*PhhA influences substrate specificity toward compounds with relatively long side chains, such as *p*-CA. As shown in Fig. 6E through H and Table S2, we hypothesized that Lys229, Leu219, Lys221, and Arg266 in *Tv*MNX3, *Tv*MNX4, *An*PhhA, and PHHY, respectively, play critical roles in defining the spatial configuration of the substrate-binding pocket among these enzymes. Similarly, 3HB4H and HQH possess basic residues (Lys247 and Arg265, respectively) at corresponding positions, suggesting that this residue is structurally conserved within the same clade of group A FPMOs (Fig. S10). To verify this hypothesis, we generated a structural model of the *Tv*MNX4_L219R variant (Fig. S13). Comparison of the variant and wild-type *Tv*MNX4 models indicated that the active-site cavity of *Tv*MNX4_L219R was smaller than that of the wild type (Fig. S13; Table S2). To further elucidate the role of this residue, the *Tv*MNX4_L219R variant was expressed and purified (Fig. S14A and B), and its catalytic activities were analyzed using 10 substrates (Fig. S14C through L). The hydroxylation efficiency and catalytic efficiency (*k*_cat_/*K*_m_) of *Tv*MNX4_L219R toward HQ and PH were higher than those of the wild-type enzyme (Table 1). In contrast, its hydroxylation and catalytic efficiencies toward *p-*CA were markedly lower than those of the wild type (Table 1). Moreover, the *Tv*MNX4_L219R was inactive toward MHQ, DMHQ, FA, and RES (Table 1). These results suggest that Leu219 in *Tv*MNX4 plays a crucial role in maintaining catalytic activity toward larger substrates.

### Transcriptional regulation of *TvMNX3* and *TvMNX4*

Previous studies have reported that transcription of *TvMNX3* is induced in the presence of 4-HBA (16). Consistent with this observation, our results showed that *TvMNX3* expression was upregulated when 4-HBA was added to the medium (Fig. S15). Additionally, when *T. versicolor* was cultured with *p*-CA, the transcription level of *TvMNX3* was 116-fold higher than that in cultures grown without the substrate (control: CON) (Fig. S15). A similar induction was observed for *Tv*MNX4 (Fig. S15). These findings suggest that both enzymes are transcriptionally regulated by aromatic compounds and are likely involved in the conversion of 4-HBA and *p*-CA in *T. versicolor*.

## DISCUSSION

**Fig 7:**
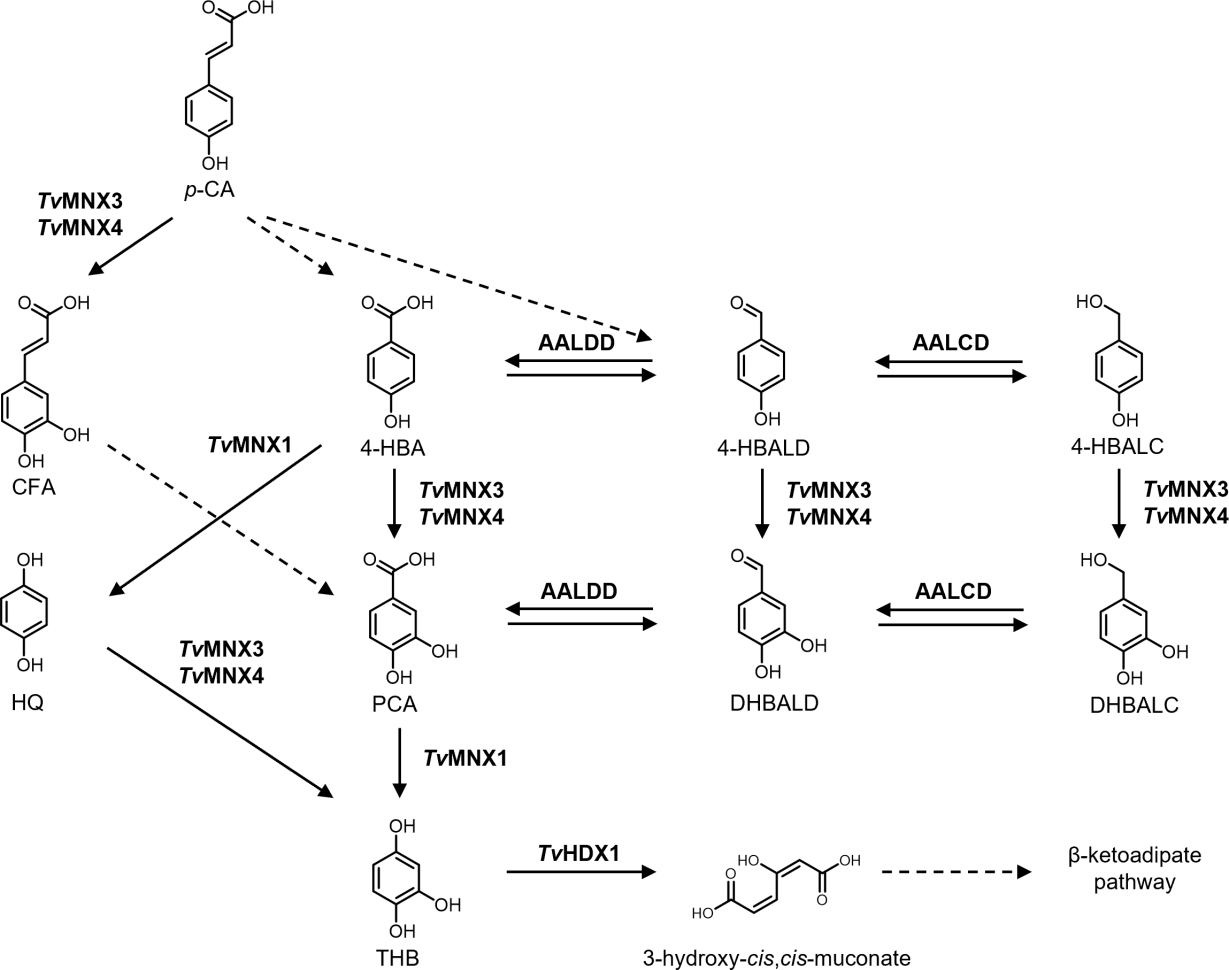
Proposed metabolic pathway of *p*-CA in the white-rot fungus *Trametes versicolor*. Dotted arrows indicate predicted reactions; solid arrows indicate enzymatically validated reactions catalyzed by FPMOs (*Tv*MNX1, *Tv*MNX3, *Tv*MNX4), aryl-aldehyde and aryl-alcohol dehydrogenases (AALDD and AALCD), and intradiol dioxygenase (*Tv*HDX1) from *T. versicolor*.

In this study, we characterized the metabolic conversion of *p*-CA by the white-rot fungus *T. versicolor. Tv*MNX3 and *Tv*MNX4 catalyzed the hydroxylation of six and ten *p*-CA derivatives and their metabolic intermediates, respectively, indicating that these enzymes likely participate in *p*-CA degradation in *T. versicolor* (Fig. 7).

*T. versicolor* initially converted *p*-CA into CFA, 4-HBA, and/or 4-HBALD, after which 4-HBALD was further oxidized to 4-HBA by arylaldehyde dehydrogenase (Fig. 7). These results indicate that *T. versicolor* employs two distinct pathways for *p*-CA degradation: (i) hydroxylation to CFA, and (ii) oxidative decarboxylation to 4-HBA and/or 4-HBALD via a CoA-dependent reaction. *p*-CA metabolism has been reported in several bacteria and fungi (14–16, 26, 27), and the pathway observed in *T. versicolor* shares common features with these systems. A recent study suggested that *p*-CA is metabolized through a CoA-dependent β-oxidation pathway to 4-HBA and/or 4-HBALD in *T. versicolor* (16). Putative enzymes catalyzing each step of the multi-step conversion from *p*-CA to 4-HBA and/or 4-HBALD have also been identified in this species (16). In this pathway, *p*-CA is activated to *p*-coumaroyl-CoA, but its subsequent conversion products vary among organisms (42, 43). In *Pseudomonas putida*, it is converted to 4-HBALD, whereas in the yeast *Rhodotorula toruloides*, it is converted to *p*-hydroxyphenyl-3-hydroxypropanoyl-CoA (42, 43). However, enzymatic validation is still required to determine whether both pathways are functionally active in *T. versicolor*. This study also demonstrated that CFA undergoes oxidative decarboxylation to form DHBALD and/or PCA (Fig. 1), suggesting that *T. versicolor* possesses enzymatic activities involved in the CoA-dependent β-oxidation of both CFA and *p*-CA (Fig. 7).

To our knowledge, this is the first report of *p*-CA hydroxylation catalyzed by group A FPMOs—*Tv*MNX3 and *Tv*MNX4—from a eukaryotic fungus. The catalytic efficiencies (*k*_cat_/*K*_m_) of *Tv*MNX4 toward *p*-CA (0.52 s**^−^**^1^ mM**^−^**^1^) and 4-HBA (0.48 s**^−^**^1^ mM**^−^**^1^) and their low degree of uncoupling of hydroxylation (6% and 8%, respectively) were comparable (Table 1), indicating that both substrates are efficiently hydroxylated by this enzyme. In contrast, *Tv*MNX3 displayed lower catalytic efficiency (*k*_cat_/*K*_m_ = 0.12 s**^−^**^1^ μM**^−^**^1^) toward *p*-CA compared with HQ (0.52 s**^−^**^1^ μM**^−^**^1^) and 4-HBA (0.42 s**^−^**^1^ μM**^−^**^1^) (Table 1). Its hydroxylation efficiency toward *p*-CA (22%) was also lower. Additionally, both enzymes hydroxylated 4-HBALD and 4-HBALC, although their hydroxylation and catalytic efficiencies were relatively low compared with those of HQ (for *Tv*MNX3) and *p*-CA (for *Tv*MNX4). The oxidative degradation pathway of 4-HBA was first reported in the ascomycetous yeast *Candida parapsilosis* (44), and the FPMOs involved were initially characterized by Eppink and colleagues (45, 46). A recent study suggested that 4-HBA undergoes oxidative decarboxylation to HQ via *Tv*MNX1 (an ortholog of *Gs*MNX1) (16), followed by hydroxylation of HQ to THB by *Tv*MNX3 in *T. versicolor* (30). THB is subsequently ring-cleaved to 3-hydroxy-*cis*,*cis*-muconate by the intradiol dioxygenase *Tv*HDX1, which then enters the β-ketoadipate pathway (30). A similar 4-HBA degradation pathway has been reported in other white-rot fungi (13). These findings suggest that the two FPMOs may participate in multiple steps of the *p*-CA catabolic pathway in *T. versicolor* (Fig. 7).

*Tv*MNX3 utilized only NADPH, whereas *Tv*MNX4 accepted both NADPH and NADH (Table 1). Given that the coenzyme specificity of PHBH and other group A enzymes can be tuned by altering the charge of the N-terminal surface loop (47–49), the presence of Lys46 in *Tv*MNX3 and Asp40 in *Tv*MNX4 may contribute to their distinct NAD(P)H preferences (Fig. S10). The positively charged Lys46 may promote NADPH-specific binding in *Tv*MNX3, whereas the negatively charged Asp40 in *Tv*MNX4 may allow recognition of both NADPH and NADH. These residues may therefore contribute to the observed differences in coenzyme recognition between *Tv*MNX3 and *Tv*MNX4. In addition, classical group A FPMOs possess a highly conserved DG sequence motif that includes residues such as Asp159, Gly160, and Arg166, which are essential for maintaining local structural integrity and supporting both FAD and NADPH binding (50). Notably, *Tv*MNX4 lacks this conserved DG motif (Fig. S10), suggesting that its cofactor-binding architecture may differ from that of typical group A enzymes.

To date, the enzymes responsible for demethylation of G (G-unit) and S (S-unit) lignin fragments have not been identified in white-rot fungi (51). Interestingly, recent studies have shown that extracellular short-chain polyphenol oxidases from lignocellulose-degrading ascomycetes can catalyze ortho-hydroxylation and oxidative demethoxylation of G- and S-type phenolic compounds, forming methoxy–ortho-diphenols (52). However, whether white-rot fungi possess similar enzymes remains unclear. Although the hydroxylation and catalytic efficiencies (*k*_cat_/*K*_m_) of *Tv*MNX4 toward MHQ, DMHQ, and FA were substantially lower than those toward *p*-CA (Table 1), the enzyme was capable of hydroxylating all three substrates. In our previous study, we demonstrated that intradiol dioxygenase 2 (IDD2) from the white-rot fungus *Phanerochaete chrysosporium* catalyzes ring cleavage of 6-methoxy-1,2,4-trihydroxybenzene (53). Conversely, methoxyhydroquinone dioxygenase (MHQD) has been reported to mediate direct ring cleavage of MHQ and DMHQ without a prior demethylation step (54). Although it remains uncertain whether IDD2 or MHQD can cleave the rings of hydroxylated derivatives such as DMHQ or FA, *Tv*MNX4 exhibited only low hydroxylation activity toward these substrates. Nevertheless, the hydroxylation of MHQ, DMHQ, and FA by *Tv*MNX4 may represent a contributing step in the degradation of G- and S-unit lignin fragments. These results suggest that *Tv*MNX3 and *Tv*MNX4 function as generalist monooxygenases capable of acting on a wide range of aromatic substrates—unlike the more substrate-specific bacterial enzymes PHBH and 3HB4H, which belong to the same clade (Fig. S1B).

Our results indicate that *p*-CA was not converted to CFA by PHHY (Fig. S8), whereas it was hydroxylated to CFA by *Tv*MNX3, *Tv*MNX4, and *An*PhhA (Table 1; Fig. S9). To explore the structural basis of these differences, we generated substrate-binding models for *p*-CA using the GNINA docking program (Fig. 5). The 4-hydroxyl group of *p*-CA formed hydrogen bonds with Asp47 and Tyr242, suggesting that Asp47 stabilizes the flavin-oxygen intermediate and facilitates substrate hydroxylation at the active site of *Tv*MNX4 (Fig. 5F). A similar binding mode was observed in *Tv*MNX3 and *An*PhhA (Fig. 5E and G), indicating that these enzymes share a conserved catalytic architecture with *Tv*MNX4. The cavity at the upper region of the substrate-binding pocket in *Tv*MNX3, *Tv*MNX4, and *An*PhhA is significantly larger than that of PHHY (Fig. 5 and 6). Among these, the cavity of *Tv*MNX4 is slightly larger than those of *Tv*MNX3 and *An*PhhA (Fig. 5 and 6), which likely accounts for the observed differences in their catalytic properties. Compared with PHHY, the larger spatial architecture of the upper active-site pocket in *Tv*MNX3, *Tv*MNX4, and *An*PhhA allows accommodation of *p*-CA, while the particularly spacious pocket of *Tv*MNX4 can also accommodate larger substrates such as RES, which contains two aromatic rings (Fig. 5 and 6). In contrast, the narrower active-site pocket in *Tv*MNX3 underlies its higher reactivity toward HQ, 4-HBALD, and 4-HBALC relative to *Tv*MNX4. Residues that define the upper-region architecture of the active-site pocket differ among *Tv*MNX3, *Tv*MNX4, *An*PhhA, and PHHY—namely, Lys229, Leu219, Lys221, and Arg266, respectively (Fig. 5 and 6). Notably, the *Tv*MNX4_L219R variant was inactive toward *p*-CA (Table 1), indicating that these residues are key determinants of pocket size and accessibility to larger substrates such as *p*-CA and RES. Collectively, these observations support the conclusion that the spatial architecture of the active-site cavity in FPMOs plays a pivotal role in defining substrate specificity.

Previous studies have reported that *p*-CA 3-hydroxylases in plants and bacteria belong to cytochrome P450s and group D FPMOs, respectively. In contrast, our findings reveal that the white-rot fungus *T. versicolor* utilizes group A FPMOs, *Tv*MNX3 and *Tv*MNX4, for the same reaction. These results indicate that *p*-CA 3-hydroxylases arise from distinct enzyme families across different species, underscoring the remarkable evolutionary diversity of this catalytic function. In addition to the group A FPMOs characterized in this study, the *T. versicolor* genome encodes 182 cytochrome P450 genes (55), some of which may also possess *p*-CA 3-hydroxylase activity. Thus, it is plausible that P450-type monooxygenases also contribute to *p*-CA conversion in *T. versicolor*.

Phenylpropanoic acids such as CFA have attracted increasing interest owing to their broad biological activities and potential use as monomers for advanced materials, including liquid crystal polymers (17–24). Similarly, piceatannol (PIC), a hydroxylated analog of resveratrol (RES), is a natural polyphenolic compound with strong antioxidant, anti-inflammatory, anticancer, and antidiabetic properties (56–58). Owing to these bioactivities, PIC represents a promising candidate for pharmaceutical and nutraceutical applications. The fungal FPMO *Tv*MNX4 catalyzes not only the hydroxylation of *p*-CA to CFA, but also the conversion of RES to PIC (Fig. S7). Microbial biosynthesis of CFA and PIC using various *p*-CA 3-hydroxylases has previously been demonstrated in engineered microbial hosts (59–62). Nevertheless, further protein engineering will likely be required to enhance the catalytic performance of *Tv*MNX4 toward RES for its application in biotechnological production systems. *Tv*MNX3 and *Tv*MNX4 thus represent promising fungal biocatalysts for the sustainable synthesis of high-value polyphenolic compounds.

In conclusion, this study demonstrates that the FPMOs *Tv*MNX3 and *Tv*MNX4 from *T. versicolor* catalyze the hydroxylation of *p*-CA, its metabolic intermediates, and the bioactive polyphenolic compound RES. To our knowledge, this is the first study to identify and characterize enzymes exhibiting *p*-CA 3-hydroxylase activity among members of the group A FPMO superfamily. Although *in vitro* assays confirmed the *p*-CA hydroxylation activities of *Tv*MNX3 and *Tv*MNX4, further genetic validation in *T. versicolor* will be essential to confirm their physiological roles *in vivo*.

## MATERIALS AND METHODS

### Chemicals and reagents

4-Hydroxybenzaldehyde (4-HBALD) and phenol (PH) were purchased from Wako Pure Chemical Industries (Osaka, Japan). *p*-Coumaric acid (*p*-CA) and its hydroxylated product, caffeic acid (CFA), together with 4-hydroxybenzoic acid (4-HBA), hydroquinone (HQ), 3,4-dihydroxybenzaldehyde (DHBALD), 4-hydroxybenzyl alcohol (4-HBALC), 3,4-dihydroxybenzyl alcohol (DHBALC), methoxyhydroquinone (MHQ), and resveratrol (RES), were obtained from Tokyo Chemical Industry Co., Ltd. (Tokyo, Japan). Protocatechuic acid (PCA) was purchased from Nacalai Tesque, Inc. (Kyoto, Japan), and 1,2,4-trihydroxybenzene (THB) was obtained from Kanto Chemical Co., Inc. (Tokyo, Japan). Ferulic acid (FA) was purchased from LKT Laboratories, Inc. (St. Paul, MN, USA), and 2,6-dimethoxyhydroquinone (DMHQ) from Apollo Scientific Ltd. (Manchester, UK). All chemicals were of analytical grade. Deionized water was prepared using a Milli-Q system (Merck Millipore, Billerica, MA, USA).

### Strains, cultures, and media

The white-rot basidiomycete *Trametes versicolor* (NBRC 30340) was maintained in high-carbon, low-nitrogen (HCLN) medium (pH 4.5) containing the following components per liter of distilled H_2_O:KH_2_PO_4_, 0.2 g; MgSO_4_⋅7H_2_O, 0.05 g; CaCl_2_, 0.01 g; mineral solution, 1 mL; and vitamin solution, 0.5 mL. The mineral solution contained (per liter of distilled H_2_O): nitrilotriacetate, 1.5 g; MgSO_4_⋅7H_2_O, 3.0 g; MnSO_4_⋅H_2_O, 0.5 g; NaCl, 1.0 g; FeSO_4_⋅7H_2_O, 100 mg; CoSO_4_, 100 mg; CaCl_2_, 82 mg; ZnSO_4_, 100 mg; CuSO_4_⋅5H_2_O, 10 mg; AlK(SO_4_)_2_, 10 mg; H_3_BO_3_, 10 mg; and NaMoO_4_, 10 mg. The vitamin solution contained (per liter of distilled H_2_O): biotin, 2 mg; folic acid, 2 mg; thiamine⋅HCl, 5 mg; riboflavin, 5 mg; pyridoxine⋅HCl, 10 mg; cyanocobalamin, 0.1 mg; nicotinic acid, 5 mg; dL-calcium pantothenate, 5 mg; *p*-aminobenzoic acid, 5 mg; and thioctic acid, 5 mg (63, 64). The medium was supplemented with 28 mM d-glucose and 1.2 mM ammonium tartrate as carbon and nitrogen sources, respectively, and buffered with 20 mM sodium 2,2-dimethylsuccinate (pH 4.5), as described previously (63). For the experiments, fungal mycelial inocula were transferred into 30 mL of HCLN medium (pH 4.5) in 300 mL Erlenmeyer flasks and incubated at 37°C under stationary conditions.

### *p*-CA metabolism by fungal cells

After a 6 day preincubation in 30 mL of HCLN medium, *p*-CA was added at a final concentration of 2 mM. After an additional 0, 2, 4, 6, or 8 days of incubation, *p*-CA and its conversion products were analyzed using an LC-20AD system (Shimadzu Corporation, Kyoto, Japan) equipped with an AQ-C18 column (250 mm × 4.6 mm i.d.; 5 µm particle size; GL Sciences Inc., Tokyo, Japan). Elution was performed with a linear gradient of distilled water and acetonitrile (100:0 → 20:80) for 5 min, followed by isocratic elution (20:80) for 5 min, at a flow rate of 1.0 mL/min.

For compound identification, culture supernatants were extracted three times with ethyl acetate, evaporated to dryness, and derivatized with *N,O*-bis(trimethylsilyl)trifluoroacetamide/pyridine (2:1, vol/vol). The trimethylsilyl (TMS) derivatives of *p*-CA and its conversion products were analyzed by GC–MS.

### Construction of the gene expression system

Full-length *Tv*MNX3 and *Tv*MNX4 genes were PCR-amplified using the primer combinations specified in Table S3. PCR reactions were performed in a DNA Thermal Cycler 2400 (Takara Bio, Otsu, Japan) with the following program: 30 cycles of denaturation at 98°C for 10 s, annealing at 55°C for 5 s, and extension at 68°C for 30 s. PCR products were separated on 1% agarose gels, stained with ethidium bromide, and visualized using a Molecular Imager FX system (Bio-Rad, Hercules, CA, USA). The amplified fragments were ligated into the EcoRI-digested pBAD/TOPO vector (Invitrogen, Carlsbad, CA, USA) using the NEBuilder HiFi DNA Assembly Master Mix (New England Biolabs, Ipswich, MA, USA). Full-length *phhy* and *AnphhA* genes were synthesized and produced by Eurofins Genomics and inserted into the same vector. Recombinant plasmids were transformed into *Escherichia coli* TOP10 (Invitrogen) using the heat-shock method, and transformants were selected on lysogeny broth agar containing ampicillin. The identity of each recombinant plasmid was confirmed by DNA sequencing. The *Tv*MNX4_L219R variant was generated by overlap extension PCR using the QuikChange Site-Directed Mutagenesis Kit (Stratagene, San Diego, CA, USA) and the primers listed in Table S3.

### Heterologous expression and purification of FPMOs

*E. coli* TOP10 cells harboring the *Tv*MNX3, *Tv*MNX4, *An*PhhA, or PHHY expression plasmids were grown at 37°C with constant shaking in Terrific broth containing 100 μg/mL ampicillin until the optical density at 600 nm reached 0.6. FPMO expression was induced by adding arabinose (final concentration, 0.02%, wt/vol), and cultures were incubated for up to 24 h at 28°C. Cells were harvested by centrifugation (3,000 × *g*, 20°C, 5 min), and pellets were resuspended in buffer A (20 mM HEPES, pH 7.4, 10% [wt/vol] glycerol, and 1 mM phenylmethylsulfonyl fluoride). Cells were lysed by sonication (5 × 30 s pulses) using a Q700 Sonicator (Qsonica, Melville, NY, USA). After centrifugation (15,000 × *g*, 4°C, 15 min) to remove insoluble debris, the supernatant was collected.

For protein purification, the crude lysate was loaded onto a nickel affinity column (Cytiva, Marlborough, MA, USA) equilibrated with buffer A at 4°C. The column was washed with buffer A, and bound proteins were eluted with a 0 M–0.3 M imidazole gradient in buffer A. Yellow fractions containing the overexpressed FPMO were pooled and further purified on a Superdex 200 HR 10/30 column (Cytiva) equilibrated with buffer A. The eluate contained the purified recombinant proteins. Protein purity was verified by SDS-PAGE. Absorption spectra were recorded using a SpectraMax spectrophotometer (Molecular Devices, San Jose, CA, USA).

### FPMO enzyme assays

FPMO activity was determined as previously described (65–67), with minor modifications. Enzyme activity and substrate specificity were analyzed in 0.5 mL reaction mixtures containing 0.1 µM enzyme, 300 μM NADPH, and 10 μL of substrate solution (0 mM–600 mM in dimethyl sulfoxide [DMSO]) in 50 mM HEPES buffer (pH 7.0 or 8.0). Reactions were initiated by substrate addition, and O_2_ consumption was monitored with a Clark-type O_2_ electrode. After incubation at 30°C for 60 min, reactions were stopped by adding 20 μL of 1 M HCl. The background O_2_ consumption rate in substrate-free controls was subtracted from the initial rates measured with substrates.

Hydroxylation efficiency (ratio of NADPH oxidation to substrate hydroxylation) was determined in 0.5 mL reactions containing 1 μM enzyme, 0.5 mM NADPH, and 0.5 mM substrate at the optimal pH for 60 min. NADPH consumption was monitored spectrophotometrically at 340 nm, and substrate hydroxylation was quantified by GC–MS.

Kinetic parameters (*K*_m_ and *k*_cat_) were calculated by fitting the initial rates to the Michaelis-Menten equation using Origin 6.0 (OriginLab, Northampton, MA, USA). Residual substrates and products were analyzed using an LC-20AD system with a linear gradient of distilled water and acetonitrile for 15 min at a flow rate of 1.0 mL/min. Reaction products were identified by GC–MS after extraction with ethyl acetate at pH 2, drying over MgSO_4_, evaporation under N_2_, and TMS derivatization, as described above. Optimal temperature was determined by measuring activity at 10°C–70°C, and optimal pH was assessed using 50 mM sodium acetate (pH 3.0–6.0), 50 mM HEPES (pH 6.0–8.5), and 50 mM Tris-HCl (pH 8.5–9.0).

### Structural analysis of *Tv*MNX3, *Tv*MNX4, and *An*PhhA

Structural models of *Tv*MNX3, *Tv*MNX4, and *An*PhhA were generated using AlphaFold2 (68) with default parameters implemented in the ColabFold environment. Ligand–docking simulations were performed using GNINA version 1.3.2 with an exhaustiveness value of 32 and the default scoring function (69). Docking conformations were visualized and analyzed using PyMOL version 2.5. The crystal structure of PHHY complexed with PH was obtained from the Protein Data Bank (PDB ID: 1PN0).

### Analytical methods

GC–MS was performed at 70 eV using a GC-MS-QP2010 system (Shimadzu, Kyoto, Japan) equipped with a 30 m fused-silica column (DB-5, J & W Scientific, Folsom, CA, USA). The oven temperature was ramped from 80°C to 320°C at 8°C/min, with an injector temperature of 280°C. Substrate-conversion products were identified by comparing GC retention times and mass-fragmentation patterns with those of authentic standards (70, 71). Compounds lacking available standards were identified by comparing their mass spectra with entries in the National Institute of Standards and Technology Mass Spectral Library.

### Quantitative RT-PCR analysis of *TvMNX3* and *TvMNX4*

After a 6 day preincubation in 30 mL of HCLN medium (pH 4.5), 4-HBA or *p*-CA dissolved in DMSO was added to a final concentration of 1 mM, and the mycelia were incubated for an additional 6 h. Total RNA was extracted from mycelia grown in the absence or presence of substrates using an RNeasy Mini Kit (Qiagen, Venlo, The Netherlands) and reverse-transcribed to synthesize cDNA. Quantitative RT-PCR was performed using *TvMNX3-* and *TvMNX4*-specific primer sets (Table S3) designed from the *T. versicolor* genome sequence, yielding products of 178 bp–226 bp. Gene expression levels were normalized to *Actin* expression (54). Real-time PCR was carried out on a Smart Cycler II (Cepheid, Sunnyvale, CA, USA) using TB Green Premix Ex Taq (Takara Bio) according to the manufacturer’s protocol. The amplification program consisted of an initial denaturation at 95°C for 30 s; 30 cycles of 95°C for 15 s; 55°C for 30 s; and 72°C for 30 s; followed by melting-curve analysis from 60°C to 95°C to verify product specificity. Relative expression levels of each gene in the presence of substrate were calculated against those in the absence of substrate.

## Data Availability

All data generated or analyzed during this study are included in this published article and its supplemental material.

## SUPPLEMENTAL MATERIAL

Supplemental material aem.02301-25-s0001.pdf Fig. S1 to S15; Tables S1 to S3.

ASM does not own the copyrights to Supplemental Material that may be linked to, or accessed through, an article. The authors have granted ASM a non-exclusive, world-wide license to publish the Supplemental Material files. Please contact the corresponding author directly for reuse.
