# Supplementary material for: Functional characterization of a novel p-coumarate 3-hydroxylase from Trametes versicolor
Source: Appl Environ Microbiol. 2026 Jan 30;92(2):e02301-25. doi: 10.1128/aem.02301-25 (PMC12915361; doi:10.1128/aem.02301-25)
Supplement: Supplemental material — Fig. S1 to S15; Tables S1 to S3. [file aem.02301-25-s0001.pdf]

Supplemental materials

Functional Characterization of a Novel *p*-Coumarate 3-Hydroxylase from *Trametes versicolor*

Running title: *p*-Coumarate 3-hydroxylase from *Trametes versicolor*

Link Hamajima, Reini Mori, Ryoga Tsurigami, Yuki Yoshida, Hiroyuki Kato, Mika Hayasaka, Hiromitsu Suzuki, Masashi Kato, and Motoyuki Shimizu#

Faculty of Agriculture, Meijo University, Nagoya, Japan

#Address correspondence to Motoyuki Shimizu (ORCID; 0000-0002-6907-6367), moshimi@meijo-u.ac.jp

A

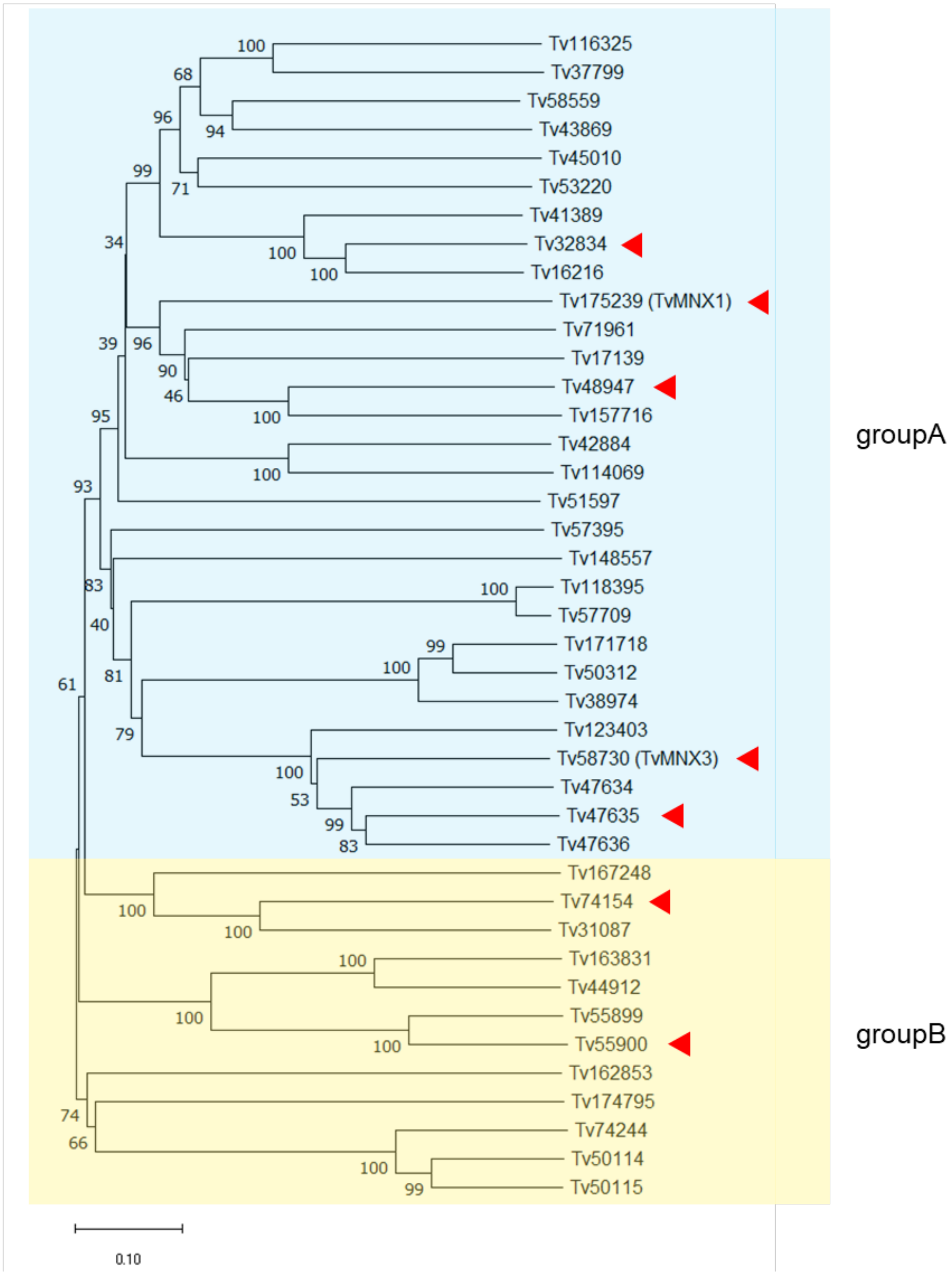
Tv116325
Tv37799
Tv58559
Tv43869
Tv45010
Tv53220
Tv41389
Tv32834
Tv16216
Tv175239 (TvMNX1)
Tv71961
Tv17139
Tv48947
Tv157716
Tv42884
Tv114069
Tv51597
Tv57395
Tv148557
Tv118395
Tv57709
Tv171718
Tv50312
Tv38974
Tv123403
Tv58730 (TvMNX3)
Tv47634
Tv47635
Tv47636
Tv167248
Tv74154
Tv31087
Tv163831
Tv44912
Tv55899
Tv55900
Tv162853
Tv174795
Tv74244
Tv50114
Tv50115
groupA
groupB
0.10

B

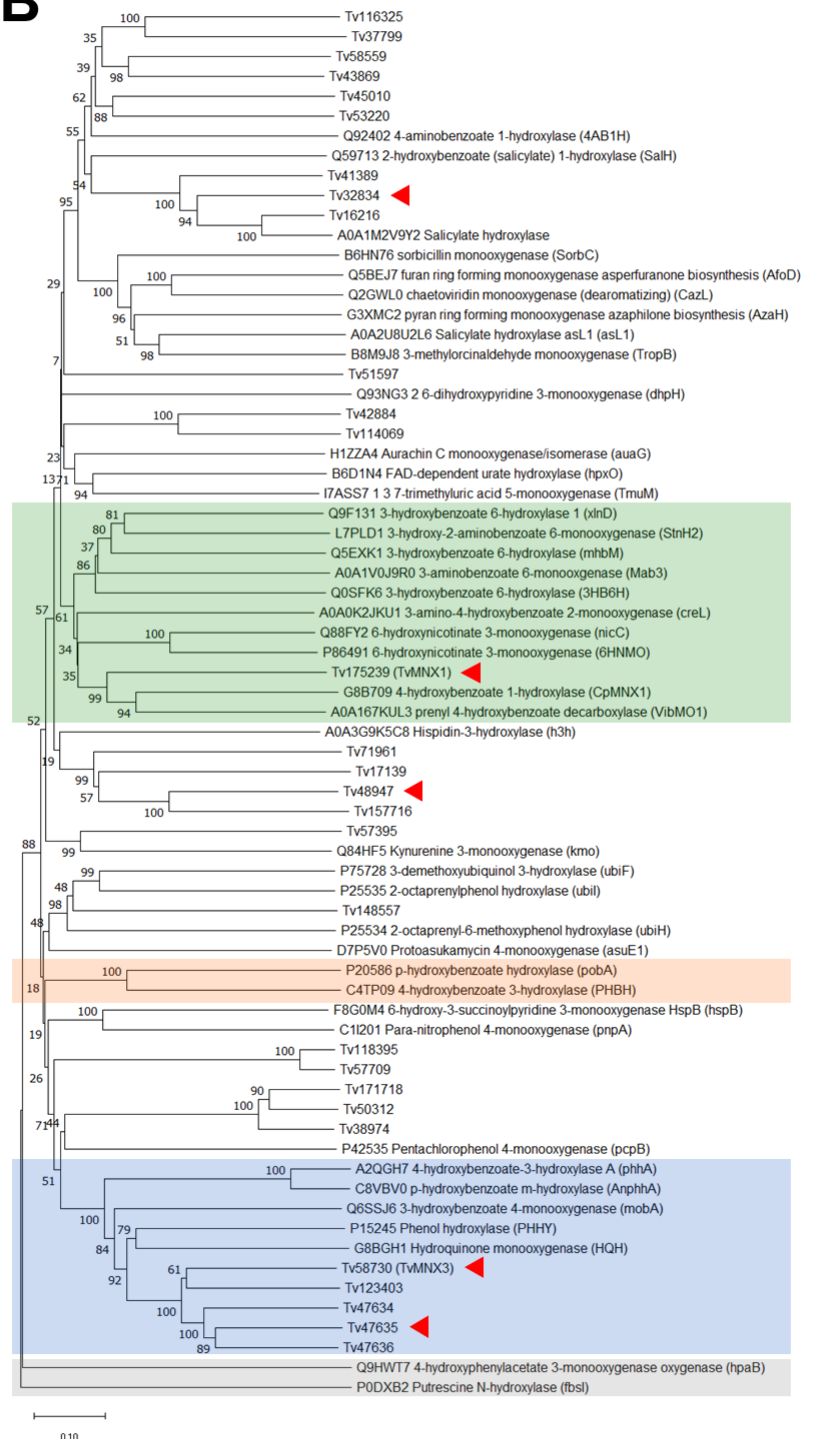
Tv116325
Tv37799
Tv58559
Tv43869
Tv45010
Tv53220
Q92402 4-aminobenzoate 1-hydroxylase (4AB1H)
Q59713 2-hydroxybenzoate (salicylate) 1-hydroxylase (SalH)
Tv41389
Tv32834
Tv16216
A0A1M2V9Y2 Salicylate hydroxylase
B6HN76 sorbicillin monooxygenase (SorbC)
Q5BEJ7 furan ring forming monooxygenase asperfuranone biosynthesis (AfoD)
Q2GWL0 chaetoviridin monooxygenase (dearomatizing) (CazL)
G3XMC2 pyran ring forming monooxygenase azaphilone biosynthesis (AzaH)
A0A2U8U2L6 Salicylate hydroxylase asL1 (asL1)
B8M9J8 3-methylorcinaldehyde monooxygenase (TropB)
Tv51597
Q93NG3 2 6-dihydroxypyridine 3-monooxygenase (dhpH)
Tv42884
Tv114069
H1ZZA4 Aurachin C monooxygenase/isomerase (auaG)
B6D1N4 FAD-dependent urate hydroxylase (hpxO)
I7ASS7 1 3 7-trimethyluric acid 5-monooxygenase (TmuM)
Q9F131 3-hydroxybenzoate 6-hydroxylase 1 (xlnD)
L7PLD1 3-hydroxy-2-aminobenzoate 6-monooxygenase (StnH2)
Q5EXK1 3-hydroxybenzoate 6-hydroxylase (mhbM)
A0A1V0J9R0 3-aminobenzoate 6-monooxgenase (Mab3)
Q0SFK6 3-hydroxybenzoate 6-hydroxylase (3HB6H)
A0A0K2JKU1 3-amino-4-hydroxybenzoate 2-monooxygenase (creL)
Q88FY2 6-hydroxynicotinate 3-monooxygenase (nicC)
P86491 6-hydroxynicotinate 3-monooxygenase (6HNMO)
Tv175239 (TvMNX1)
G8B709 4-hydroxybenzoate 1-hydroxylase (CpMNX1)
A0A167KUL3 prenyl 4-hydroxybenzoate decarboxylase (VibMO1)
A0A3G9K5C8 Hispidin-3-hydroxylase (h3h)
Tv71961
Tv17139
Tv48947
Tv157716
Tv57395
Q84HF5 Kynurenine 3-monooxygenase (kmo)
P75728 3-demethoxyubiquinol 3-hydroxylase (ubiF)
P25535 2-octaprenylphenol hydroxylase (ubiI)
Tv148557
P25534 2-octaprenyl-6-methoxyphenol hydroxylase (ubiH)
D7P5V0 Protoasukamycin 4-monooxygenase (asuE1)
P20586 p-hydroxybenzoate hydroxylase (pobA)
C4TP09 4-hydroxybenzoate 3-hydroxylase (PHBH)
F8G0M4 6-hydroxy-3-succinoylpyridine 3-monooxygenase HspB (hspB)
C1I201 Para-nitrophenol 4-monooxygenase (pnpA)
Tv118395
Tv57709
Tv171718
Tv50312
Tv38974
P42535 Pentachlorophenol 4-monooxygenase (pcpB)
A2QGH7 4-hydroxybenzoate-3-hydroxylase A (phhA)
C8VBV0 p-hydroxybenzoate m-hydroxylase (AnphhA)
Q6SSJ6 3-hydroxybenzoate 4-monooxygenase (mobA)
P15245 Phenol hydroxylase (PHHY)
G8BGH1 Hydroquinone monooxygenase (HQH)
Tv58730 (TvMNX3)
Tv123403
Tv47634
Tv47635
Tv47636
Q9HWT7 4-hydroxyphenylacetate 3-monooxygenase oxygenase (hpaB)
P0DXB2 Putrescine N-hydroxylase (fbsI)
0.10

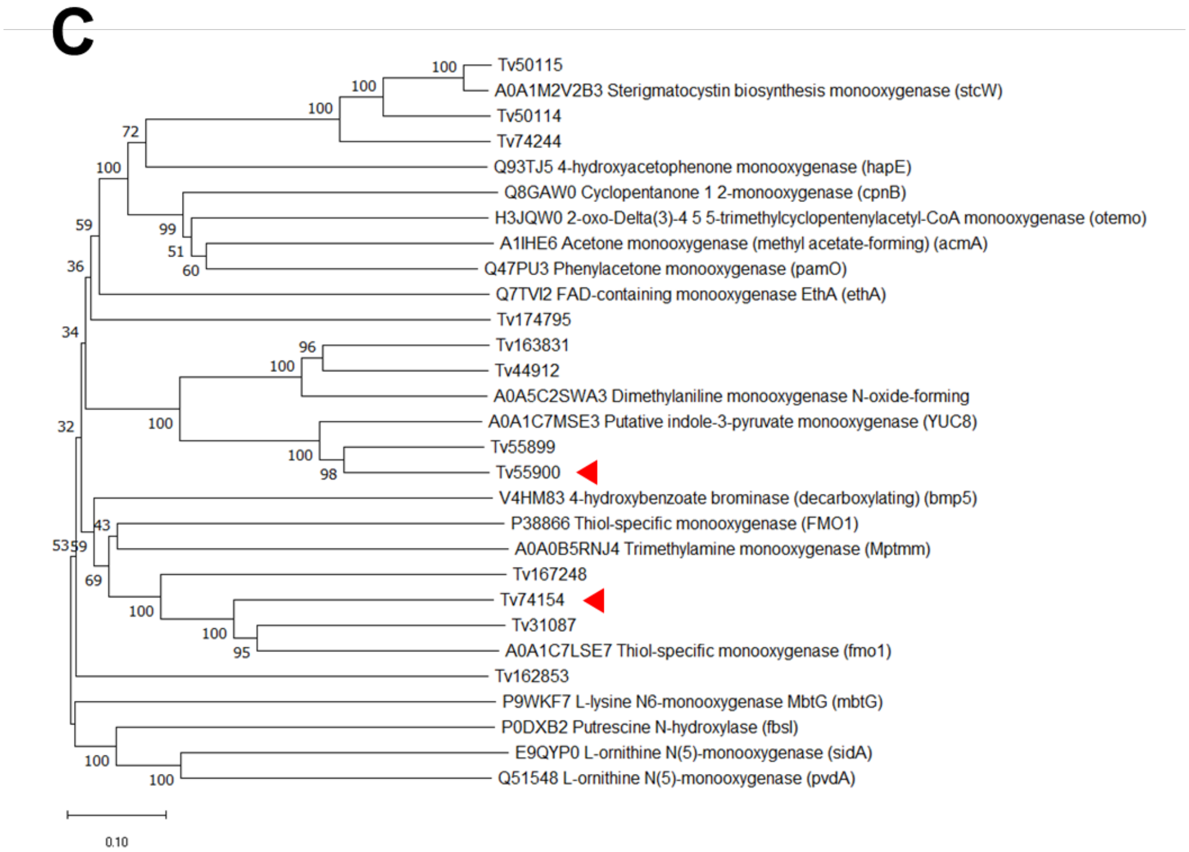

FIG. S1 Phylogenetic analysis of flavoprotein monooxygenases (FPMOs) from *Trametes versicolor*.

(A) Bootstrap consensus tree of 41 FPMO protein sequences identified in the *T. versicolor* genome (https://mycocosm.jgi.doe.gov/trave1/trave1.home.html). Twenty-nine sequences were assigned to group A and twelve to group B FPMOs.

(B) Bootstrap consensus tree of 29 *T. versicolor* FPMOs together with biochemically characterized group A FPMOs. Three clades of group A enzymes are color-coded according to the classification described in reference (31). Only enzymes with experimentally validated or well-defined functional annotations were included to improve interpretability

of the phylogeny. Representative outgroup sequences from group B and group D FPMOs were included.

(C) Bootstrap consensus tree of 12 *T. versicolor* FPMOs together with 17 biochemically characterized group B FPMOs.

Sequences with available UniProt IDs are indicated. Arrowheads denote the recombinant enzymes prepared and characterized in this study (*Tv*MNX1, *Tv*MNX3, *Tv*47635, *Tv*32834, *Tv*48947, *Tv*55900, and *Tv*74154).

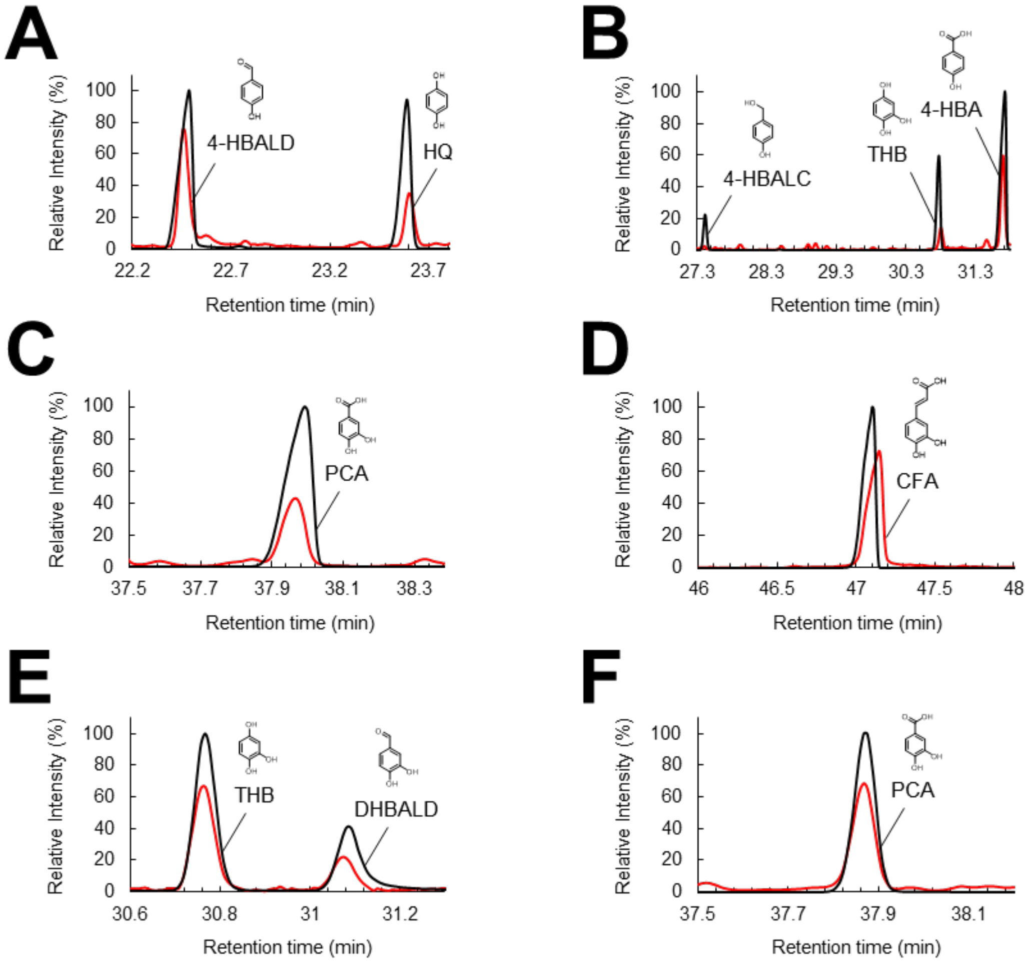

FIG. S2 Total ion chromatograms of metabolites derived from *p*-CA and CFA. (A–D) Total ion chromatograms of trimethylsilyl (TMS)-derivatized metabolites produced from *p*-coumaric acid (*p*-CA). (E, F) Total ion chromatograms of TMS-derivatized metabolites produced from caffeic acid (CFA). Analyses were performed using gas chromatography–mass spectrometry (GC–MS). Peaks corresponding to caffeic acid (CFA), 4-hydroxybenzoic acid (4-HBA), protocatechuic acid (PCA), hydroquinone (HQ), 1,2,4-trihydroxybenzene (THB), 4-hydroxybenzaldehyde (4-HBALD), 4-hydroxybenzyl alcohol (4-HBALC), and 3,4-dihydroxybenzaldehyde (DHBALD) appeared at retention times of 47.1, 31.7, 37.9, 23.6, 30.7, 22.5, 27.4, and 31.1 min, respectively. Each experiment was

performed three times, and representative chromatograms are shown. Black traces indicate chromatograms of authentic standards.

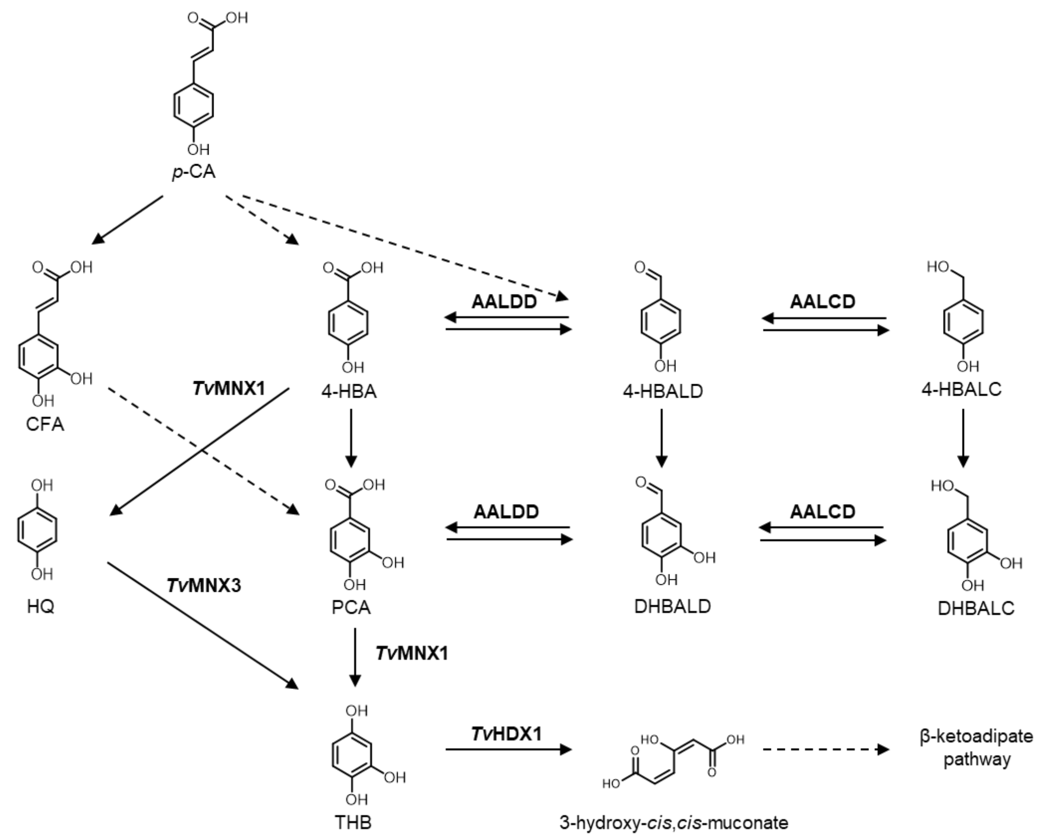

FIG. S3 Proposed metabolic pathway of *p*-coumaric acid (*p*-CA) in the white-rot fungus *Trametes versicolor*.

Dotted arrows indicate predicted reactions; solid arrows indicate enzymatically validated reactions catalyzed by flavoprotein monooxygenases (*Tv*MNX1, *Tv*MNX3), aryl-aldehyde and aryl-alcohol dehydrogenases (AALDD and AALCD), and intradiol dioxygenase (*Tv*HDX1) from *T. versicolor*.

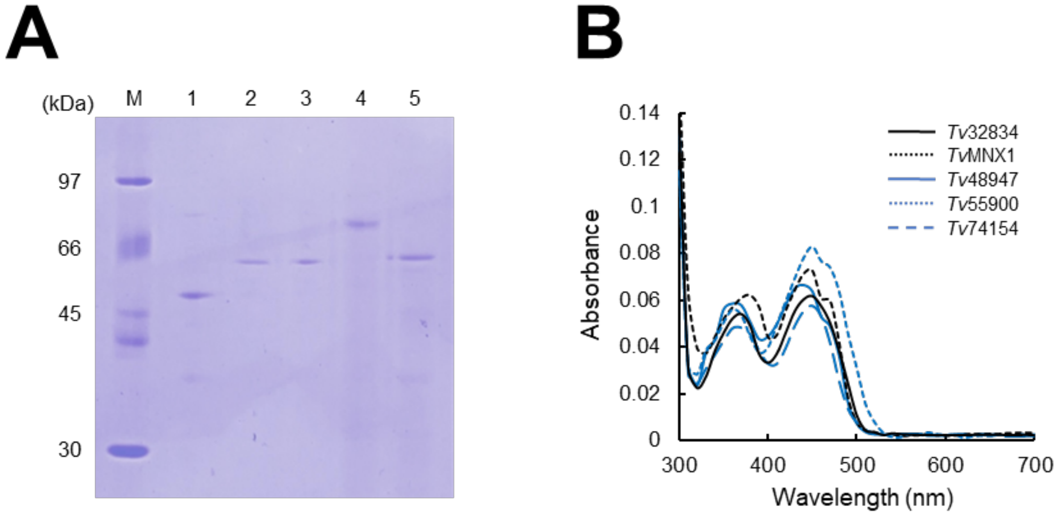

FIG. S4 SDS–PAGE analysis and absorption spectra of recombinant *Tv*MNXs.

(A) SDS–PAGE analysis of purified *Tv*MNXs. Lane 1, *Tv*32834; lane 2, *Tv*MNX1; lane 3, *Tv*48947; lane 4, *Tv*55900; lane 5, *Tv*74154; lane M, molecular-mass marker. (B) UV–visible absorption spectra of purified *Tv*MNXs.

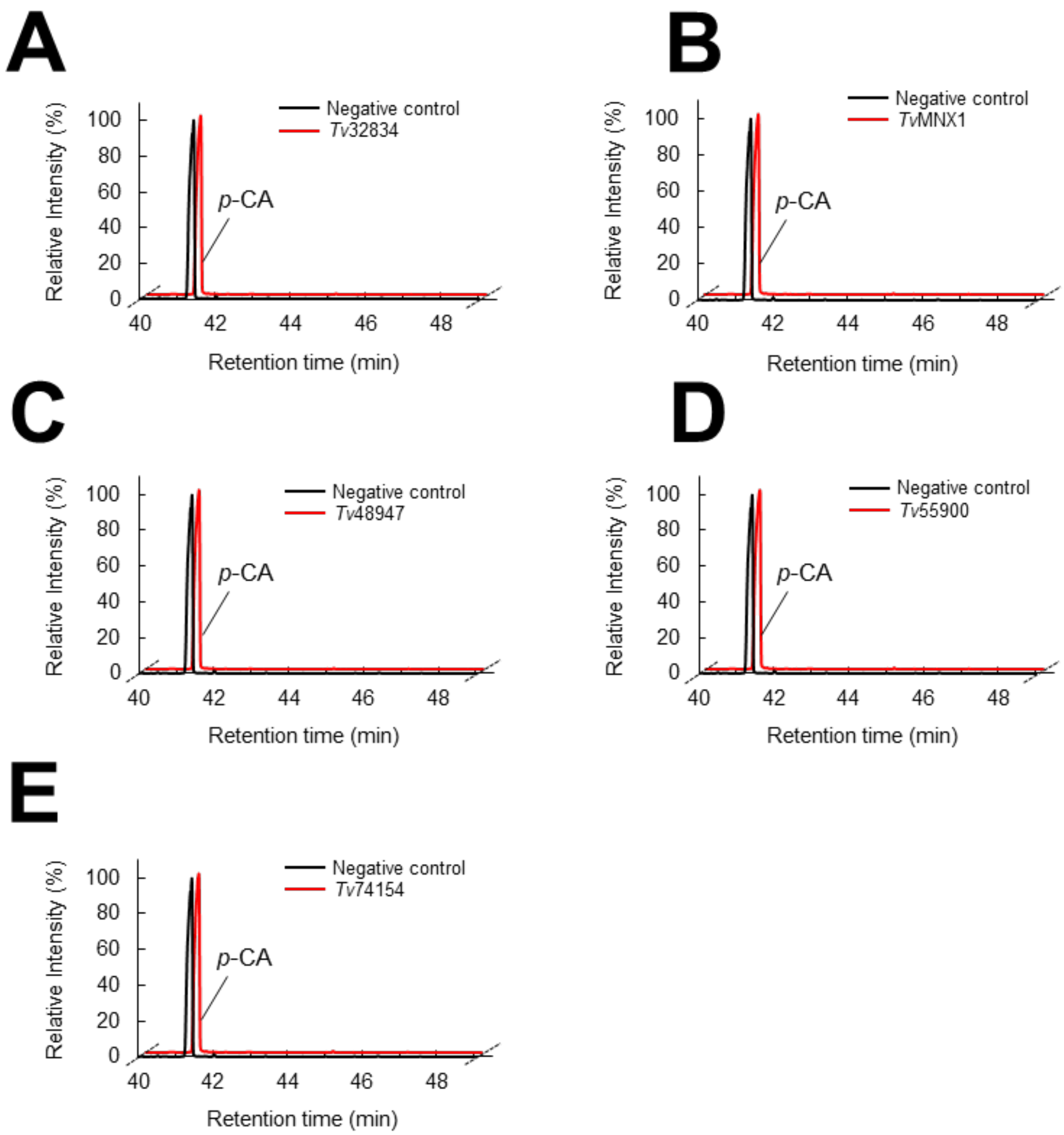

FIG. S5 Total ion chromatograms of reaction products generated by recombinant FPMOs from *p*-CA.

(A–E) Total ion chromatograms of trimethylsilyl (TMS)-derivatized reaction products generated from *p*-coumaric acid (*p*-CA) by *Tv*32834 (A), *Tv*MNX1 (B), *Tv*48947 (C), *Tv*55900 (D), and *Tv*74154 (E). The products were analyzed by gas chromatography–mass spectrometry (GC–MS). Each experiment was performed three times, and representative chromatograms are shown.

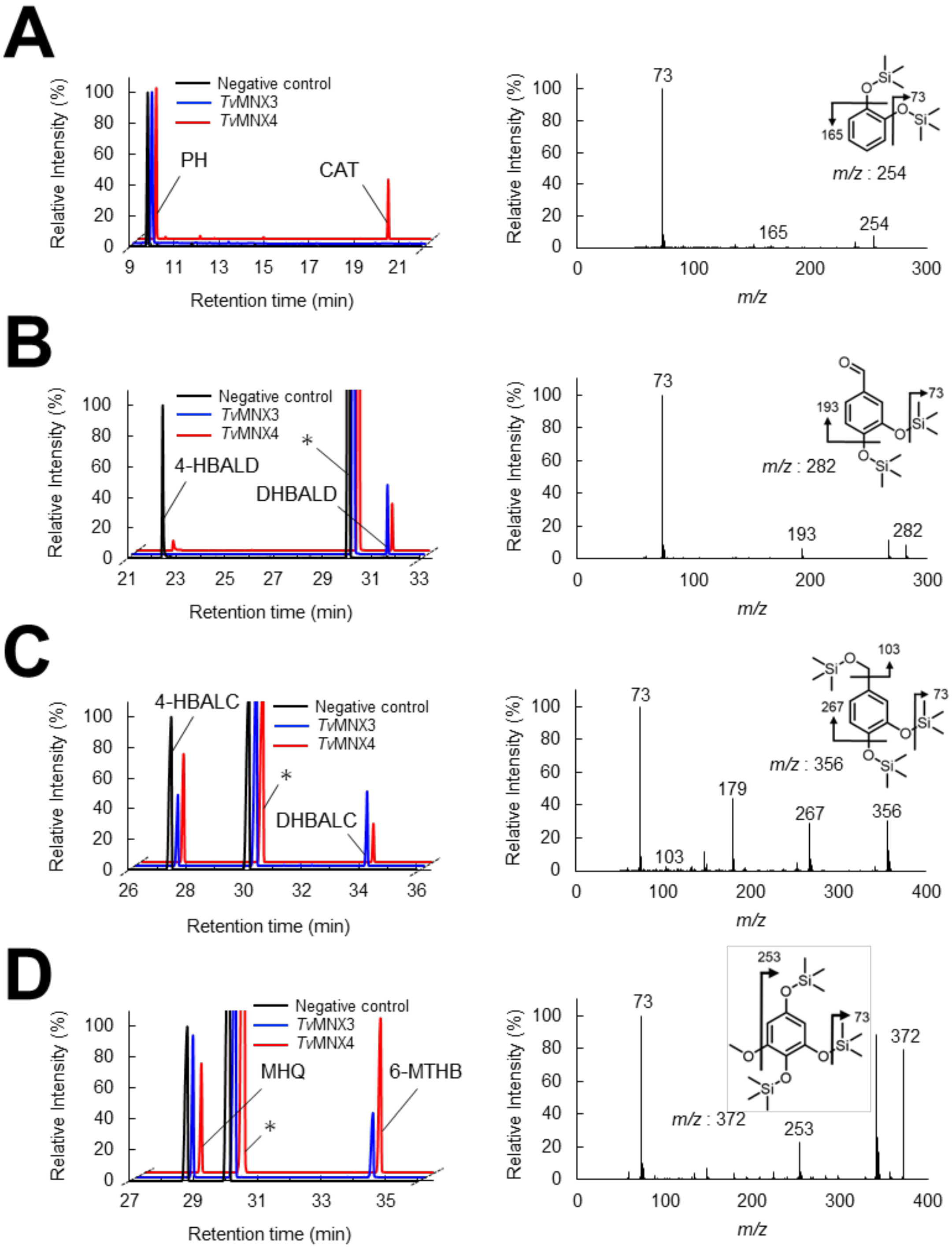

A
Negative control
TvMNX3
TvMNX4
PH
CAT
Relative Intensity (%)
Retention time (min)
m/z : 254
73
165
254
m/z
B
4-HBALD
DHBALD
*
m/z : 282
193
282
C
4-HBALC
DHBALC
m/z : 356
103
179
267
356
D
MHQ
6-MTHB
m/z : 372
253
372

77

78

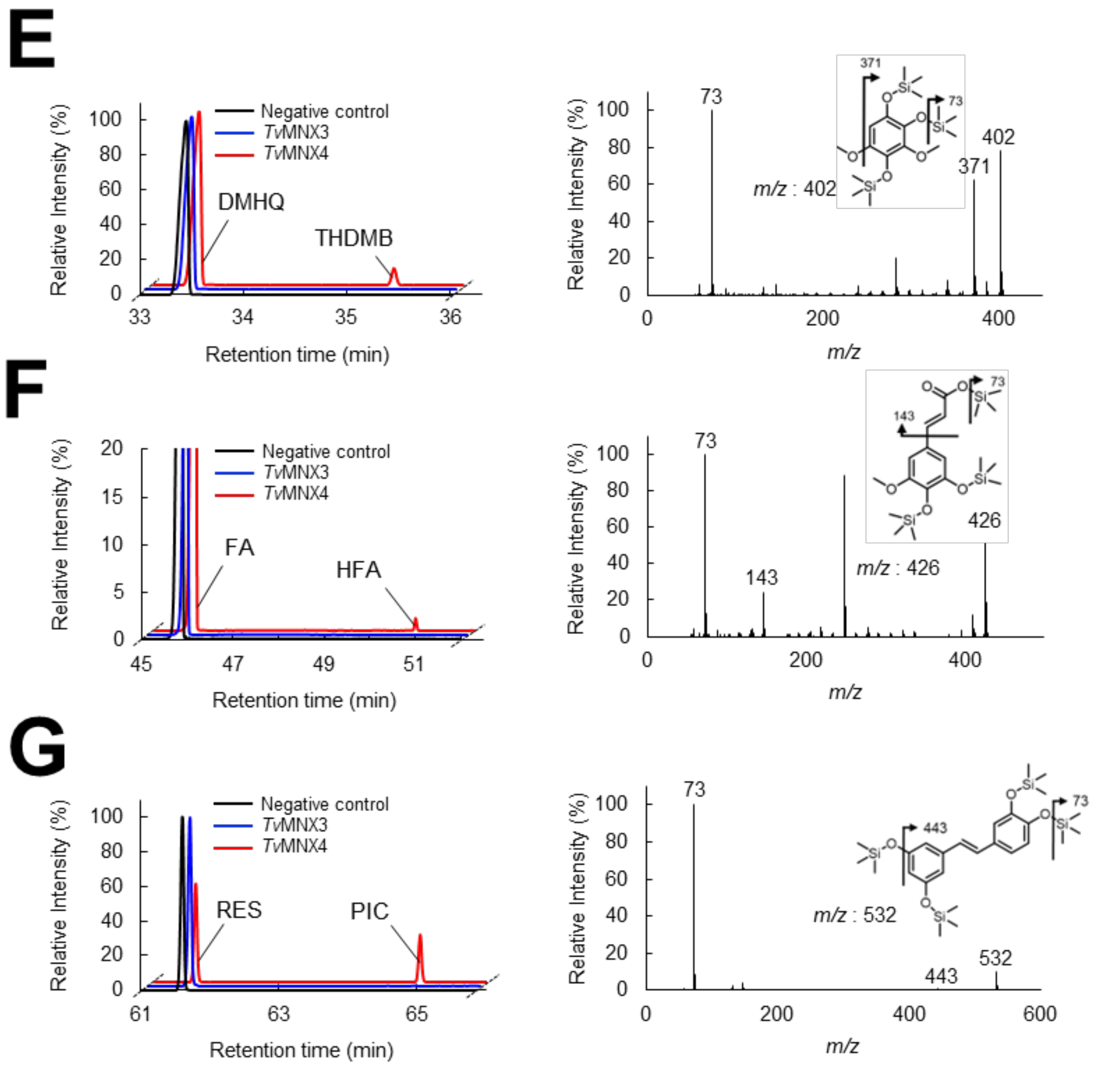

FIG. S6 Total ion chromatograms and mass spectra of reaction products generated by *Tv*MNX3 and *Tv*MNX4.

Reaction products were generated using phenol (PH) (A), 4-hydroxybenzaldehyde (4-HBALD) (B), 4-hydroxybenzyl alcohol (4-HBALC) (C), 6-methoxyhydroquinone (MHQ) (D), 2,6-dimethoxyhydroquinone (DMHQ) (E), ferulic acid (FA) (F), and resveratrol (RES) (G) as substrates. Trimethylsilyl (TMS)-derivatized products were analyzed by GC–MS. Mass spectra corresponding to (A) catechol (CAT), (B) 3,4-dihydroxybenzaldehyde (DHBALD), (C) 3,4-dihydroxybenzyl alcohol (DHBALC), (D) 6-methoxy-1,2,4-

trihydroxybenzene (6-MTHB), (E) 1,2,4-trihydroxy-3,5-dimethoxybenzene (THDMB), (F) 5-hydroxyferulic acid (HFA), and (G) piceatannol (PIC) were obtained from GC peaks at retention times of 20.1, 31.3, 34.0, 34.3, 35.3, 50.6, and 64.8 min, respectively. Asterisks indicate contaminants. Each experiment was performed three times, and representative data are shown.

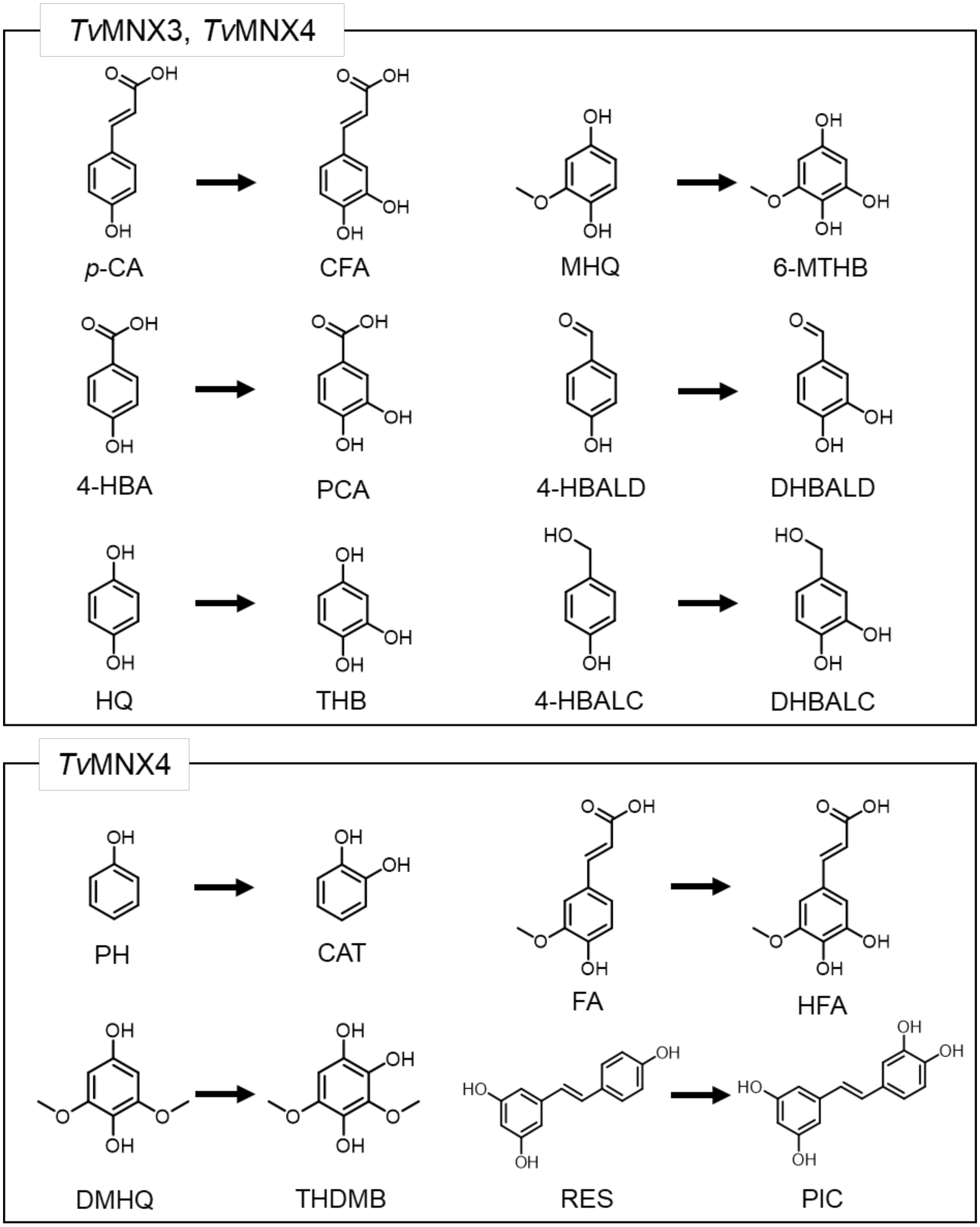

FIG. S7 Hydroxylation of six and ten aromatic substrates by *Tv*MNX3 and *Tv*MNX4. *Tv*MNX3 and *Tv*MNX4 catalyzed the hydroxylation of six and ten aromatic compounds, respectively. Representative substrates include *p*-coumaric acid (*p*-CA), 4-hydroxybenzoic acid (4-HBA), 4-hydroxybenzaldehyde (4-HBALD), 4-hydroxybenzyl alcohol (4-

HBALC), hydroquinone (HQ), 1,2,4-trihydroxybenzene (THB), ferulic acid (FA), methoxyhydroquinone (MHQ), 2,6-dimethoxyhydroquinone (DMHQ), and resveratrol (RES). The figure summarizes the structural diversity of the substrates and their hydroxylated products identified by gas chromatography–mass spectrometry (GC–MS).

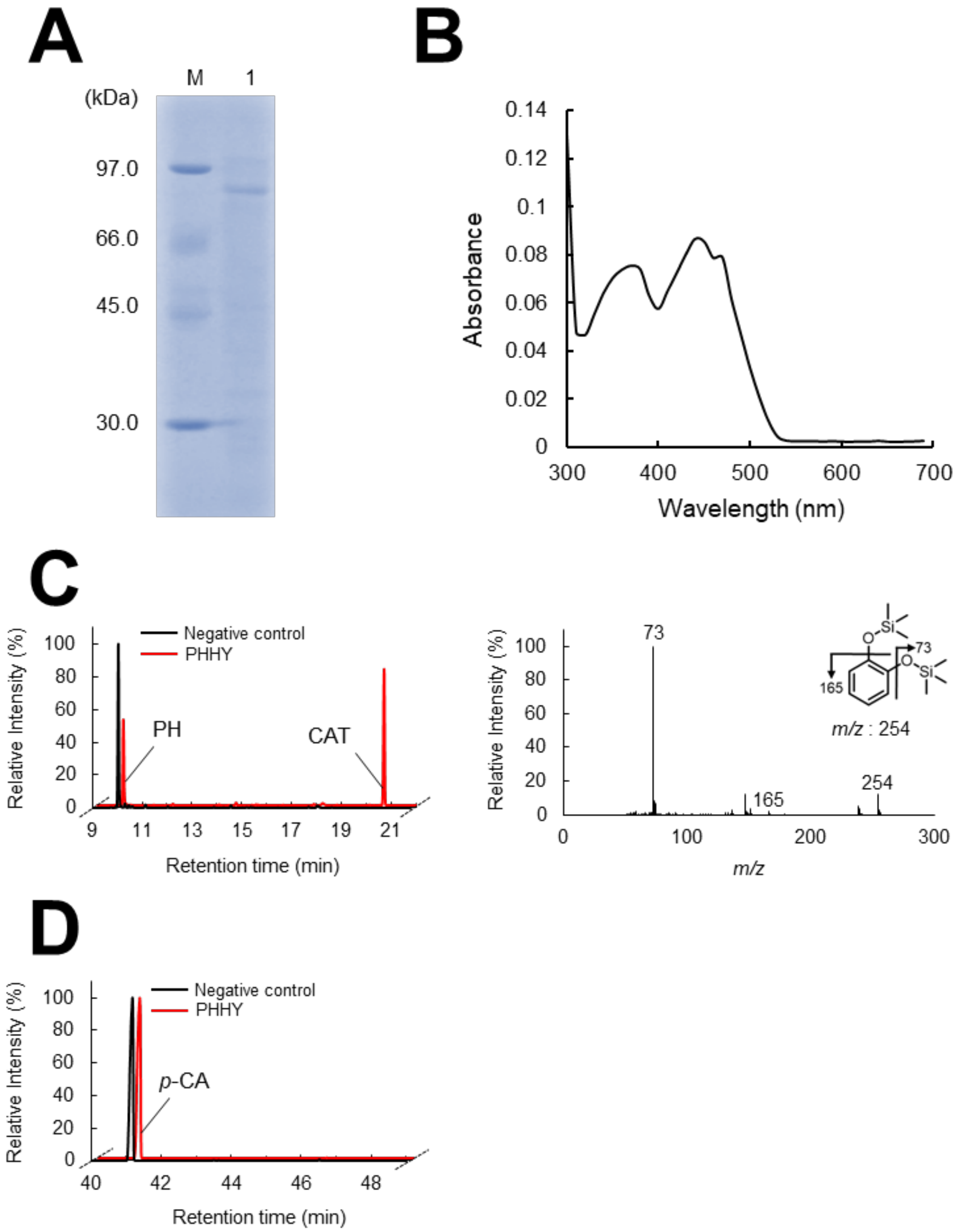

FIG. S8 Preparation and catalytic characterization of recombinant PHHY.

(A) SDS–PAGE analysis of purified PHHY. Lane 1, PHHY; lane M, molecular-mass marker. (B) UV–visible absorption spectrum of purified PHHY. (C, D) Total ion chromatograms and mass spectra of trimethylsilyl (TMS)-derivatized reaction products

generated by PHHY from phenol (PH) (C) and *p*-coumaric acid (*p*-CA) (D) as substrates. The TMS-derivatized product from PH appeared at a retention time of 20.5 min. Each experiment was performed three times, and representative chromatograms are shown.

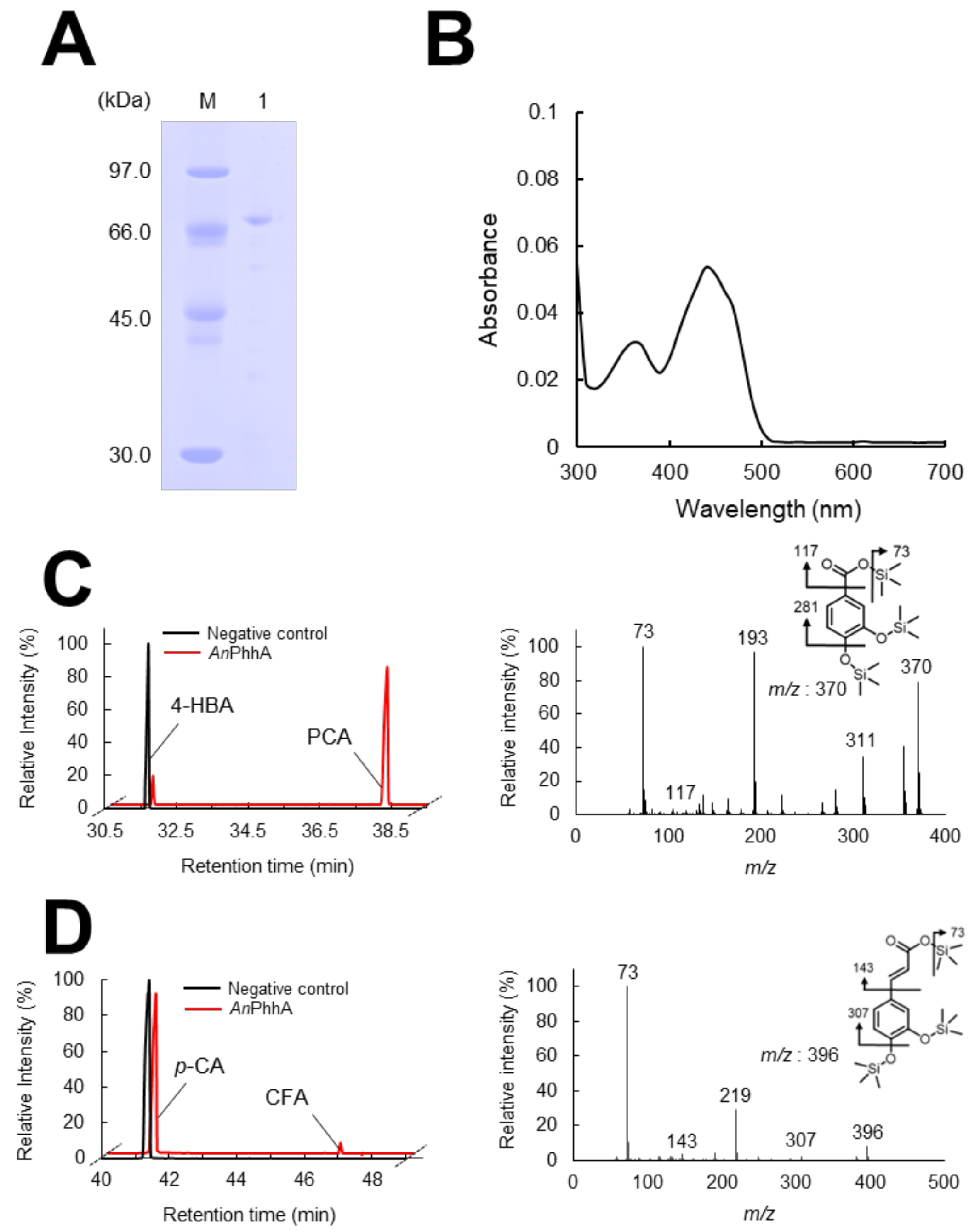

FIG. S9 Preparation and catalytic characterization of recombinant *An*PhhA.

(A) SDS–PAGE analysis of purified *An*PhhA. Lane 1, *An*PhhA, lane M, molecular-mass marker. (B) UV–visible absorption spectrum of purified *An*PhhA. (C, D) Total ion chromatograms and mass spectra of trimethylsilyl (TMS)-derivatized reaction products generated by *An*PhhA from 4-hydroxybenzoic acid (4-HBA) (C) and *p*-coumaric acid (*p*-

CA) (D) as substrates. The TMS-derivatized products appeared at retention times of 38.1 min (C) and 47.0 min (D). Each experiment was performed three times, and representative chromatograms are shown.

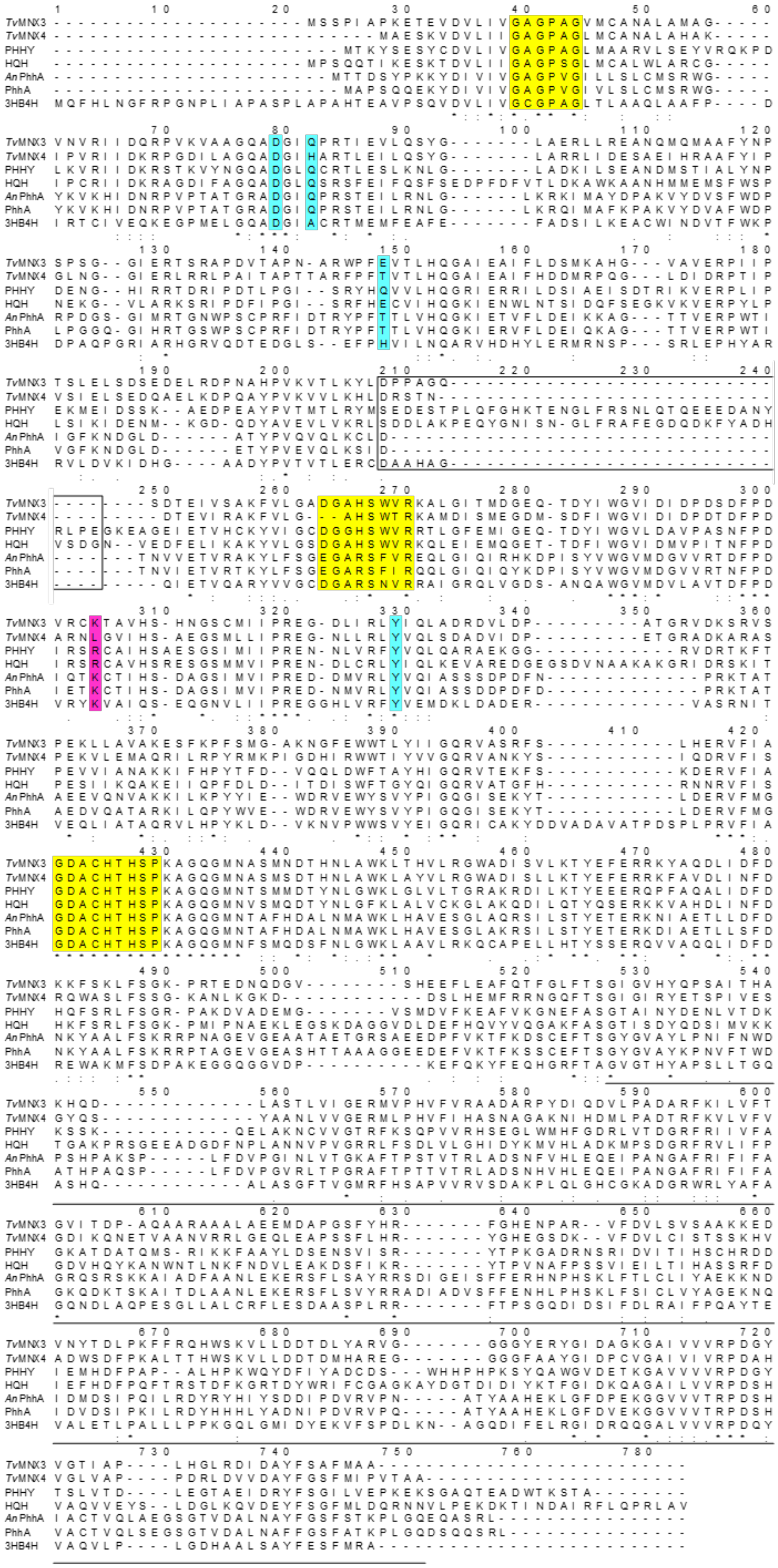
TvMNX3
TvMNX4
PHHY
HQH
AnPhhA
PhhA
3HB4H

Fig. S10 Multiple sequence alignment of representative group A FPMOs.

The alignment includes *Tv*MNX3 and *Tv*MNX4 from *Trametes versicolor*, PHHY from *Trichosporon cutaneum*, HQH from *Candida parapsilosis*, *An*PhhA from *Aspergillus nidulans*, PhhA from *Aspergillus niger* and 3HB4H from *Comamonas testosteroni*, all belonging to the same clade shown in Fig. S1B.

FAD-binding motifs (fingerprints) are highlighted in yellow; conserved active-site residues are shown in blue. Boxed regions denote active-site loops, and amino acid residues shaping the upper architecture of the active-site pocket are highlighted in pink. The underlined region represents an additional thioredoxin-like domain specific to this clade.

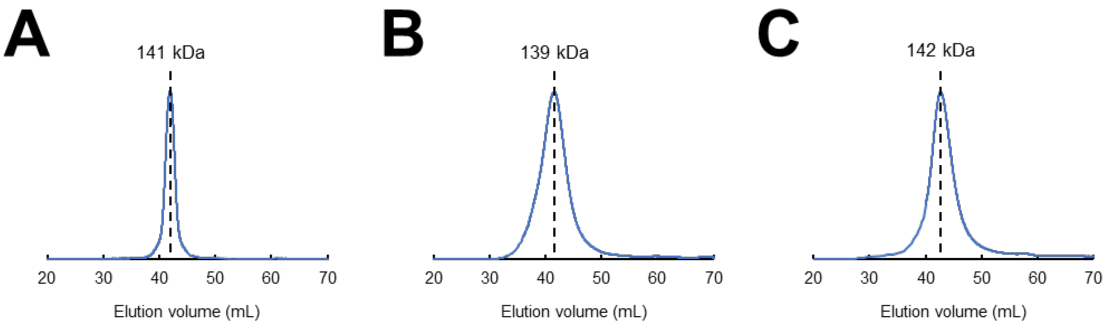

FIG. S11 Determination of molecular mass of *Tv*MNX3, *Tv*MNX4, and *An*PhhA.

Purified flavoprotein monooxygenases were analyzed by gel-filtration chromatography. The estimated molecular mass is indicated by a dashed line.

(A–C) Elution profiles of *Tv*MNX3 (A), *Tv*MNX4 (B), and *An*PhhA (C).

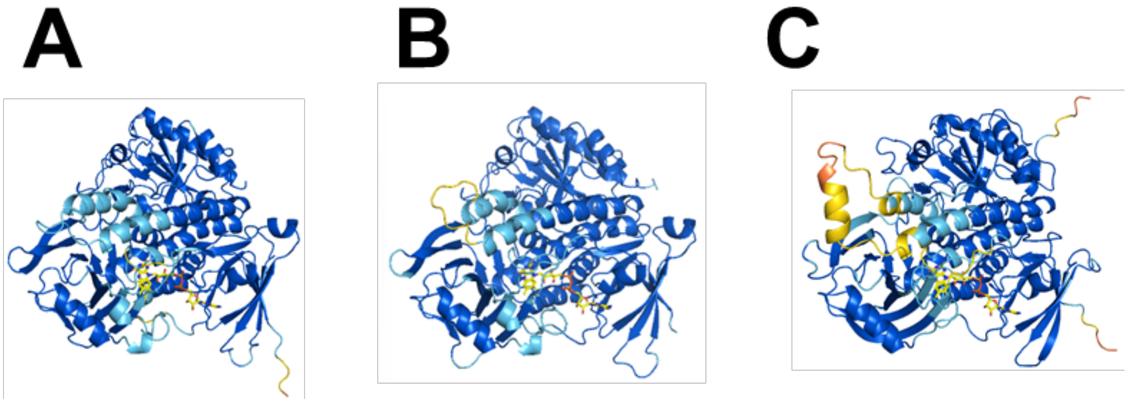

FIG. S12 Structural predictions of *Tv*MNX3, *Tv*MNX4, and *An*PhhA.

(A–C) Closed-conformation models of *Tv*MNX3 (A), *Tv*MNX4 (B), and *An*PhhA (C) predicted using AlphaFold2. Model confidence (pLDDT) is color-coded: navy blue (>90), cyan (70–90), yellow (50–70), and orange (<50). Bound flavin adenine dinucleotide (FAD) is shown as a yellow stick.

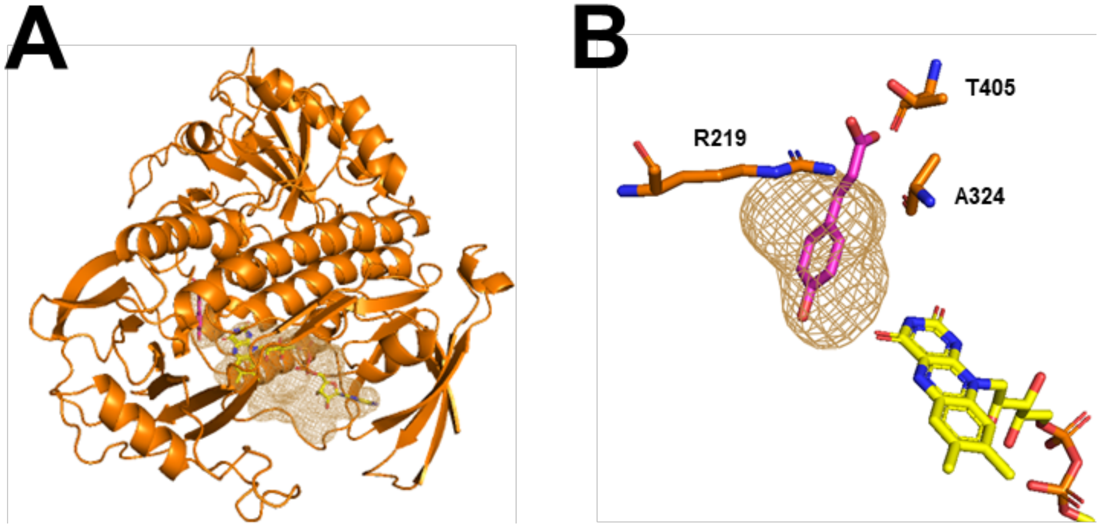

FIG. S13 Structural prediction of the *Tv*MNX4_L219R variant.

(A, B) Structural models of the *Tv*MNX4_L219R variant. (A) Closed-conformation model of the *Tv*MNX4_L219R variant docked with *p*-coumaric acid (*p*-CA), predicted using AlphaFold2 and the GNINA docking program. (B) Cavity in the active-site pocket of *Tv*MNX4_L219R with *p*-CA represented as a brown mesh. Magenta and yellow sticks denote the substrate (*p*-CA) and the cofactor flavin adenine dinucleotide (FAD), respectively.

191

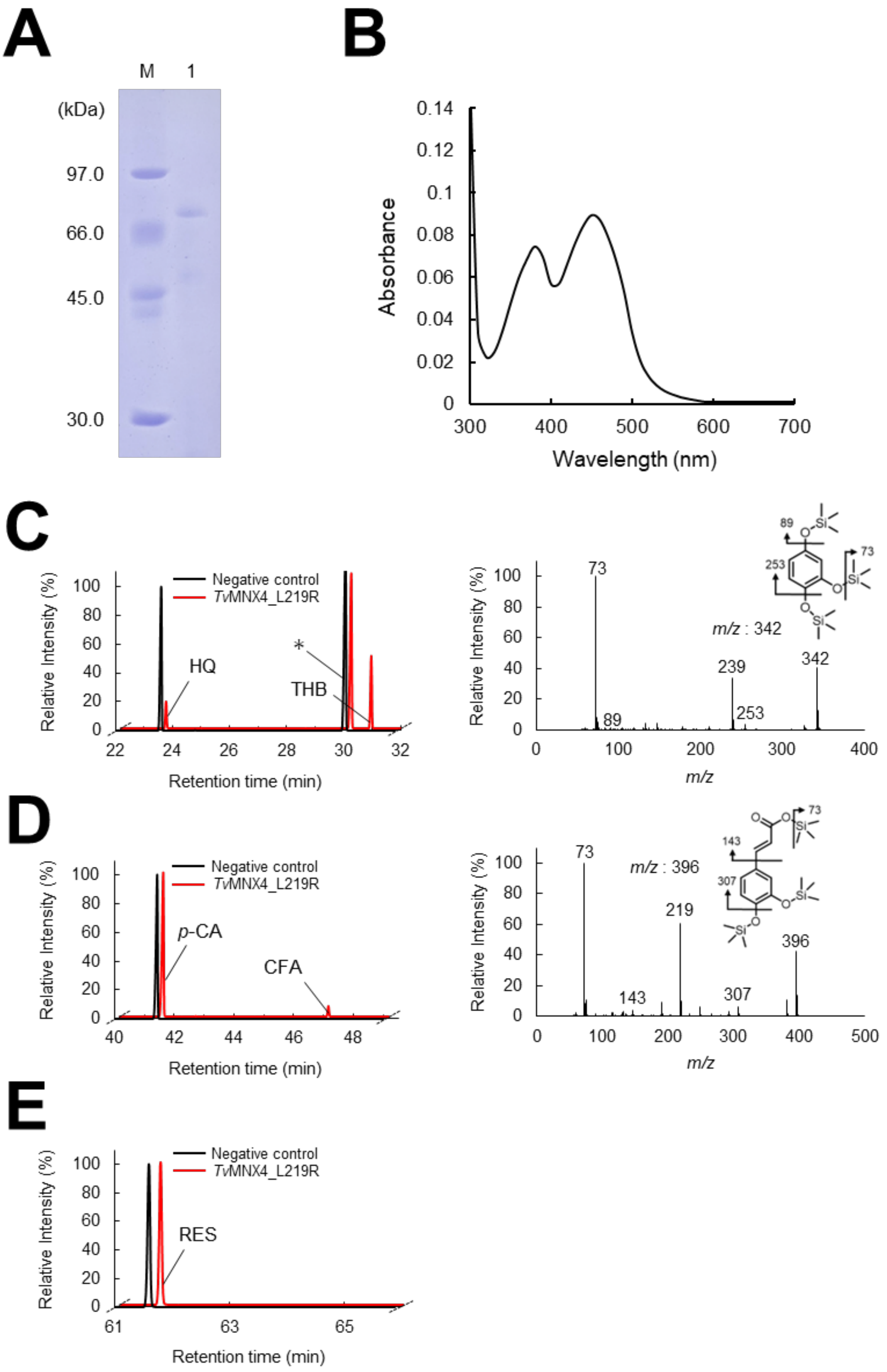

A
M
1
(kDa)
97.0
66.0
45.0
30.0
B
Absorbance
0.14
0.12
0.1
0.08
0.06
0.04
0.02
0
300
400
500
600
700
Wavelength (nm)
C
Negative control
TvMNX4_L219R
Relative Intensity (%)
HQ
*
THB
Retention time (min)
m/z : 342
73
89
239
253
342
m/z
D
p-CA
CFA
m/z : 396
73
143
219
307
396
E
RES

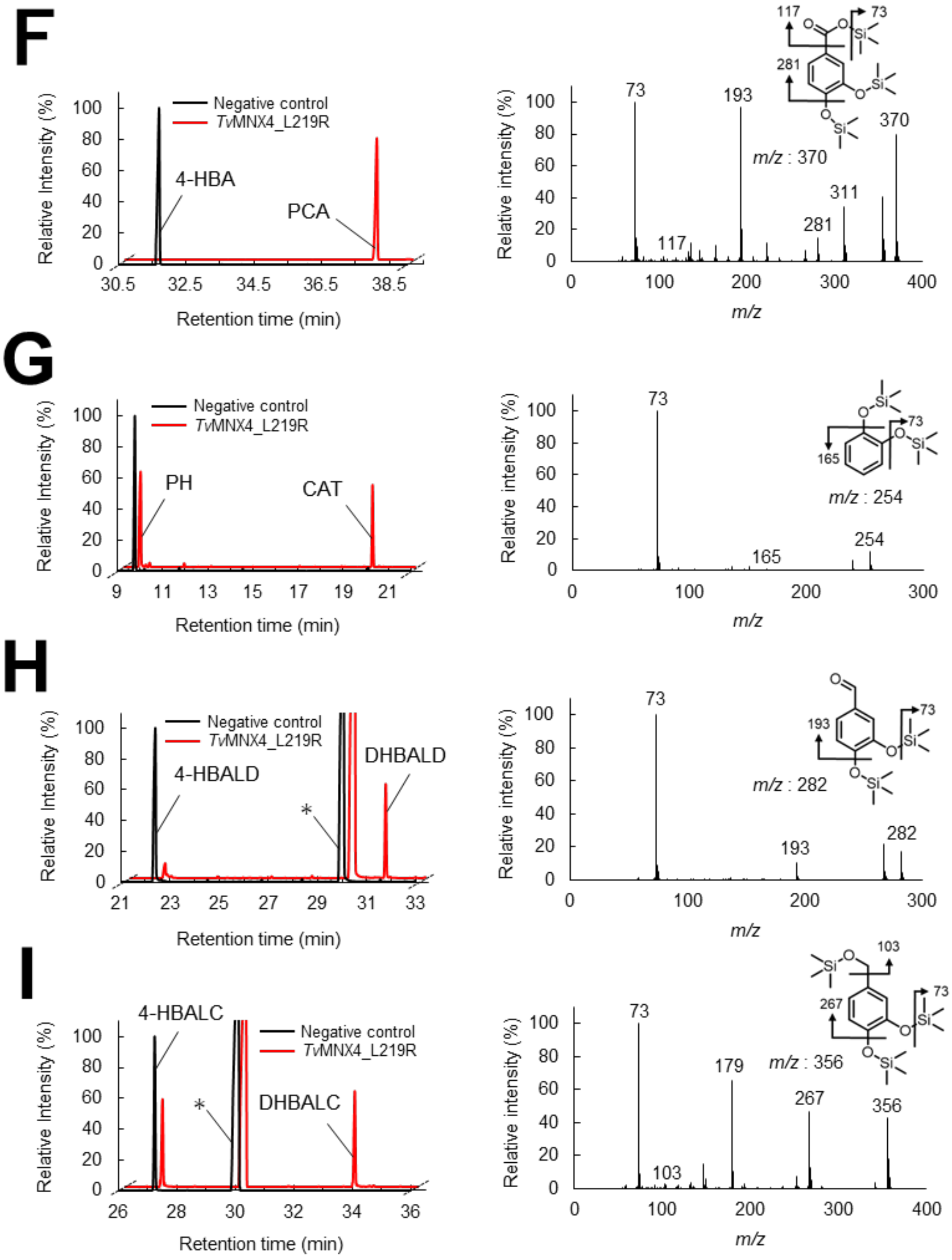
F
Negative control
TvMNX4_L219R
4-HBA
PCA
Relative Intensity (%)
Retention time (min)
Relative intensity (%)
m/z
m/z : 370
73
117
193
281
311
370
G
PH
CAT
165
254
m/z : 254
H
4-HBALD
DHBALD
*
m/z : 282
282
I
4-HBALC
DHBALC
m/z : 356
103
179
267
356

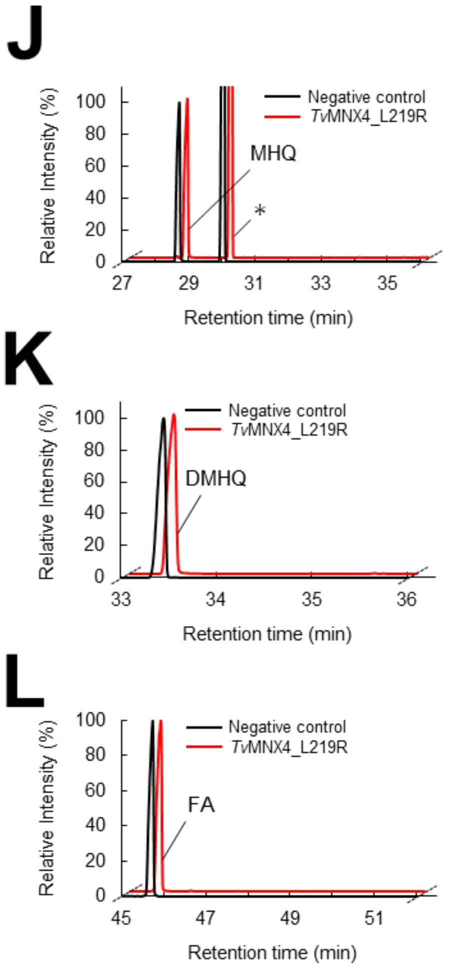

FIG. S14 Preparation and catalytic characterization of the recombinant *Tv*MNX4_L219R variant.

(A) SDS–PAGE analysis of the purified *Tv*MNX4_L219R variant. Lane 1, *Tv*MNX4_L219R; lane M, molecular-mass marker.

(B) UV–visible absorption spectrum of the *Tv*MNX4_L219R variant.

(C–L) Total ion chromatograms and mass spectra of trimethylsilyl (TMS)-derivatized reaction products generated by *Tv*MNX4_L219R from hydroquinone (HQ) (C), *p*-coumaric acid (*p*-CA) (D), resveratrol (RES) (E), 4-hydroxybenzoic acid (4-HBA) (F), phenol (PH) (G), 4-hydroxybenzaldehyde (4-HBALD) (H), 4-hydroxybenzyl alcohol (4-HBALC) (I), methoxyhydroquinone (MHQ) (J), 2,6-dimethoxyhydroquinone (DMHQ)

(K), and ferulic acid (FA) (L). Reaction products were analyzed by gas chromatography–mass spectrometry (GC–MS). Mass spectra of representative products—(C) 1,2,4-trihydroxybenzene (THB), (D) caffeic acid (CFA), (F) protocatechuic acid (PCA), (G) catechol (CAT), (H) 3,4-dihydroxybenzaldehyde (DHBALD), and (I) 3,4-dihydroxybenzyl alcohol (DHBALC)—were obtained from GC peaks at retention times of 30.7, 46.9, 38.0, 20.0, 31.3, and 34.0 min, respectively. Each experiment was performed three times, and representative chromatograms are shown.

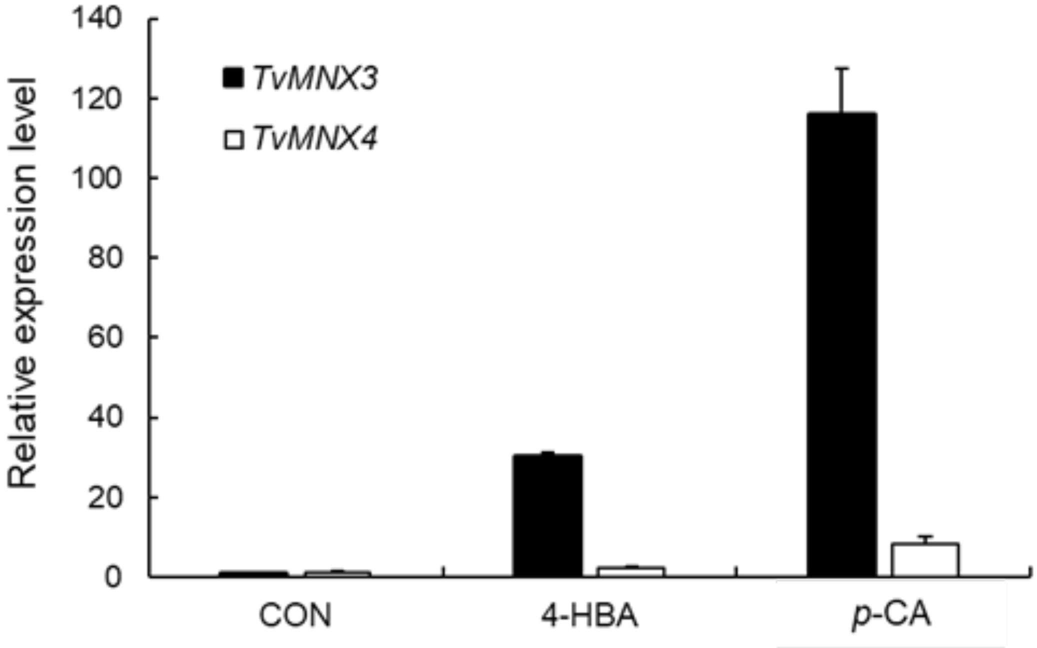

FIG. S15 Gene expression profiles of *TvMNX3* and *TvMNX4* in response to exogenous 4-HBA and *p*-CA.

Transcript abundances of *Tv*MNX3 and *Tv*MNX4 were normalized against the *ACT1* reference gene. Bars represent normalized expression levels following 6 h of exposure to 1 mM 4-hydroxybenzoic acid (4-HBA) or *p*-coumaric acid (*p*-CA) relative to untreated controls. Data represent the mean ± standard deviation (error bars) from three independent experiments.

Table S1 Theoretical and experimental molecular masses, protein yields, and A_{280}/A_{450} ratios of seven recombinant flavoprotein monooxygenases from *Trametes versicolor*.

	Theoretical MWs (kDa)	Experimental MWs (kDa)	Yield of purified FPMOs (mg/L culture)	Theoretical A280/A450	Experimental A280/A450
*Tv*MNX3	70.1	69.1	55.6	6.30	6.51
*Tv*MNX4	69.7	70.4	61.2	6.53	6.81
*Tv*32834	50.0	50.5	33.2	5.13	6.20
*Tv*MNX1	55.8	59.3	49.2	5.76	6.00
*Tv*48947	55.4	59.3	30.4	4.85	5.31
*Tv*55900	71.2	73.7	40.4	11.8	12.6
*Tv*74154	58.2	62.6	26.8	8.07	10.8

Recombinant *T. versicolor* flavoprotein monooxygenase (FPMO) candidates were expressed in *Escherichia coli* and purified. Theoretical molecular masses were calculated from the amino acid sequences, including the C-terminal 6×His tag, whereas experimental molecular masses were estimated by SDS–PAGE (Fig. 2 and Fig. S4). Protein yields (mg/L culture) were determined from the total amount of purified protein obtained after Ni-affinity chromatography. Theoretical A_{280}/A_{450} ratios were calculated using the predicted extinction coefficients at 280 nm derived from amino acid composition and the experimentally determined extinction coefficient of flavin adenine dinucleotide (FAD) at 450 nm (ε_{450} = 11.3 mM^{-1} cm^{-1}). In these calculations, the contribution of protein-bound FAD to absorbance at 280 nm was explicitly taken into account and subtracted to obtain corrected theoretical A_{280}/A_{450} values. Experimental A_{280}/A_{450} ratios were obtained from the UV–visible absorption spectra of the purified enzymes.

Table S2 Calculated cavity volumes at the upper region of the active-site pockets of fungal flavoprotein monooxygenases belonging to the same clade.

FPMO	*Tv* MNX3	*Tv* MNX4	*Tv* MNX4_L219R	PHHY	*An* PhhA	HQH
Cavity volume ($Å^3$)	124	176	113	60	115	146

Cavity volumes were calculated from the structural models of *Tv*MNX3, *Tv*MNX4, *Tv*MNX4_L219R, PHHY, *An*PhhA, and HQH using PyMOL.

Table S3 Oligonucleotide primers used for recombinant protein expression.

Gene-specific primers were designed based on genomic sequence data available from the DOE Joint Genome Institute (*Trametes versicolor*; https://mycocosm.jgi.doe.gov/trave1/trave1.home.html) and UniProt database entries corresponding to the target genes.

Primer	Gene	Nucleotide sequence
**Cloning for recombinant protein production**		
Tv MNX3-f	*Tv* 58730	5'-GAGATATACATACCCATGTCGTCCCCTATTGCAC-3'
Tv MNX3-r		5'-CCTTCAAGCTCGCCCTTTGCTGCCATGAACGCG-3'
Tv MNX4-f	*Tv* 47635	5'-GAGATATACATACCCATGGCGGAAAGTAAGGTCG-3'
Tv MNX4-r		5'-CCTTCAAGCTCGCCCTTCGCAGCGGTGACTGGG-3'
Tv 32834-f	*Tv* 32834	5'-GAGATATACATACCCATGCAGCAGACACACCAGA-3'
Tv 32834-r		5'-CCTTCAAGCTCGCCCTTTGCACTGAACGCCCCTC-3'
Tv MNX1-f	*Tv* 175239	5'-GAGATATACATACCCATGACCAGCCACCAACCCA-3'
Tv MNX1-r		5'-CCTTCAAGCTCGCCCTTCAGCTTGTGCTCCACC-3'
Tv 48947-f	*Tv* 48947	5'-GAGATATACATACCCATGAGTGCTGAATCATCTA-3'
Tv 48947-r		5'-CCTTCAAGCTCGCCCTTCTCGTCGTAGTCAGTG-3'
Tv 55900-f	*Tv* 55900	5'-GAGATATACATACCCATGGCCGCCGCGACCCACAC-3'
Tv 55900-r		5'-CCTTCAAGCTCGCCCTTCACATAGCGCTCCTCG-3'
Tv 74154-f	*Tv* 74154	5'-GAGATATACATACCCATGGCCTCCTCTTCCACAA-3'
Tv 74154-r		5'-CCTTCAAGCTCGCCCTTCTCCTCCAGCGGCGCC-3'
PHHY-f	*phhy*	5'-AGGAGAAATTAACCATGGGAGGATCCACCAAATATAGTGAAAGCTA-3'
PHHY-r	(UniProt ID : P15245)	5'-TGGTGATGGTGATGAGATCTGGATCCTGCGGTGCTTTTGGTCCAGT-3'
An PhhA-f	*An* 3445	5'-GAGATATACATACCCATGACGACCGATAGTTAC-3'
An PhhA-r	(UniProt ID : 10952)	5'-CTTCAAGCTCGCCCTTCAGACGGCTAGCTTGC-3'
**Site-directed mutagenesis**		
Tv MNX4_L219R-f	*Tv* 47635	5'-CCCGACGCCCGAAATCGAGGTGTCATCCACTC-3'
Tv MNX4_L219R-r		5'-GAAGTCTGTATCGGGGTCGATGTCTATCACACC-3'
**Real time PCR**		
RT *Tv* MNX3-f	*Tv* 58730	5'-CCTACCTCCCTGGAGCTATC-3'
RT *Tv* MNX3-r		5'-CGATCACGCCCCAGATGTAG-3'
RT *Tv* MNX4-f	*Tv* 47635	5'-ACCCTCAGGCCTATCCAGTG-3'
RT *Tv* MNX4-r		5'-GGGTCGATGTCTATCACACC-3'
RT *Tv* ACT-f	*Tv* 25778	5'-CCGAAATGCTTCTTTCCGTC-3'
RT *Tv* ACT-r		5'-CCCAACTCTTCTGCATACAC-3'
